# Incidence of Diabetes in Children and Adolescents During the COVID-19 Pandemic: A Systematic Review and Meta-Analysis

**DOI:** 10.1001/jamanetworkopen.2023.21281

**Published:** 2023-06-30

**Authors:** Daniel D’Souza, Jessica Empringham, Petros Pechlivanoglou, Elizabeth M. Uleryk, Eyal Cohen, Rayzel Shulman

**Affiliations:** 1 Child Health Evaluative Sciences, SickKids Research Institute, Toronto, Ontario, Canada; 2 Department of Pediatrics, University of Toronto, Toronto, Ontario, Canada; 3 Institute of Health Policy, Management and Evaluation, University of Toronto, Toronto, Ontario, Canada; 4 E.M. Uleryk Consulting, Mississauga, Ontario, Canada; 5 Edwin S.H. Leong Centre for Healthy Children, University of Toronto, Toronto, Ontario, Canada; 6 Division of Endocrinology, Hospital for Sick Children, Toronto, Ontario, Canada

## Abstract

**Importance:**

There are reports of increasing incidence of pediatric diabetes since the onset of the COVID-19 pandemic. Given the limitations of individual studies that examine this association, it is important to synthesize estimates of changes in incidence rates.

**Objective:**

To compare the incidence rates of pediatric diabetes during and before the COVID-19 pandemic.

**Data Sources:**

In this systematic review and meta-analysis, electronic databases, including Medline, Embase, the Cochrane database, Scopus, and Web of Science, and the gray literature were searched between January 1, 2020, and March 28, 2023, using subject headings and text word terms related to COVID-19, diabetes, and diabetic ketoacidosis (DKA).

**Study Selection:**

Studies were independently assessed by 2 reviewers and included if they reported differences in incident diabetes cases during vs before the pandemic in youths younger than 19 years, had a minimum observation period of 12 months during and 12 months before the pandemic, and were published in English.

**Data Extraction and Synthesis:**

From records that underwent full-text review, 2 reviewers independently abstracted data and assessed the risk of bias. The Meta-analysis of Observational Studies in Epidemiology (MOOSE) reporting guideline was followed. Eligible studies were included in the meta-analysis and analyzed with a common and random-effects analysis. Studies not included in the meta-analysis were summarized descriptively.

**Main Outcomes and Measures:**

The primary outcome was change in the incidence rate of pediatric diabetes during vs before the COVID-19 pandemic. The secondary outcome was change in the incidence rate of DKA among youths with new-onset diabetes during the pandemic.

**Results:**

Forty-two studies including 102 984 incident diabetes cases were included in the systematic review. The meta-analysis of type 1 diabetes incidence rates included 17 studies of 38 149 youths and showed a higher incidence rate during the first year of the pandemic compared with the prepandemic period (incidence rate ratio [IRR], 1.14; 95% CI, 1.08-1.21). There was an increased incidence of diabetes during months 13 to 24 of the pandemic compared with the prepandemic period (IRR, 1.27; 95% CI, 1.18-1.37). Ten studies (23.8%) reported incident type 2 diabetes cases in both periods. These studies did not report incidence rates, so results were not pooled. Fifteen studies (35.7%) reported DKA incidence and found a higher rate during the pandemic compared with before the pandemic (IRR, 1.26; 95% CI, 1.17-1.36).

**Conclusions and Relevance:**

This study found that incidence rates of type 1 diabetes and DKA at diabetes onset in children and adolescents were higher after the start of the COVID-19 pandemic than before the pandemic. Increased resources and support may be needed for the growing number of children and adolescents with diabetes. Future studies are needed to assess whether this trend persists and may help elucidate possible underlying mechanisms to explain temporal changes.

## Key Points

**Question:**

Was there a change in the incidence of diabetes in children and adolescents after the onset of the COVID-19 pandemic?

**Findings:**

In this systematic review and meta-analysis of 42 studies including 102 984 youths, the incidence of type 1 diabetes was higher during the COVID-19 pandemic compared with before the pandemic.

**Meaning:**

The findings suggest the need to elucidate possible underlying mechanisms to explain temporal changes and increased resources and support for the growing number of children and adolescents with diabetes.

## Teaser

This systematic review and meta-analysis compares incidence rates of diabetes and diabetic ketoacidosis among children and adolescents before and during the COVID-19 pandemic.

## Introduction

Diabetes is a common chronic disease in children.^1,2^ Several studies have reported an increased incidence of types 1 and 2 diabetes in children since the COVID-19 pandemic.^3,4^ Some studies reported an association between SARS-CoV-2 infection and new-onset diabetes.^5,6^ However, given the challenges of ascertaining a SARS-CoV-2 infection, there are concerns about the validity of these studies. Furthermore, there is no clear mechanism by which COVID-19 could directly or indirectly lead to new-onset type 1 or 2 diabetes.^7^ The pathophysiology of types 1 and 2 diabetes are distinct, as are the theoretical pathways by which COVID-19 might cause them^8^; therefore, it is important to determine whether there has been an increased incidence rate of 1 or both types of diabetes.

The examination of diabetes incidence rates during the pandemic is nuanced because there was a preexisting increase of 3% to 4% in the annual incidence rate of type 1 diabetes reported in European countries,^9^ seasonality to diabetes incidence,^10,11^ and variability in the reported incidence rates between early and later months during the pandemic.^12,13^ It is important to establish whether the reported increased incidence rates of new-onset diabetes in children are overall higher and sustained or a result of a catch-up effect from a lower incidence rate early in the pandemic likely due to delays in diagnoses.^7,14^

A recent review and meta-analysis^4^ that pooled results of 8 studies reported that the incidence rate of type 1 diabetes was higher during the pandemic in 2020 (32.39 per 100 000 children) compared with the same period prior to the pandemic in 2019 (19.73 per 100 000 children). An important limitation of that meta-analysis is that it only included studies conducted during the first wave of the pandemic. There may have been a lower incidence rate early in the pandemic and a higher incidence rate later in the pandemic due, in part, to the absence of an expected seasonal decline in summer months.^12^ Importantly, the meta-analysis^4^ only examined the incidence rate of type 1 diabetes in children. It is plausible that the increase in sedentary behavior observed during the COVID-19 pandemic due to school closures and lockdown measures was associated with the increased prevalence of childhood obesity, a known risk factor for type 2 diabetes.^15,16^ In addition to reports of an increased incidence rate of diabetes, there have also been consistent reports of an increased risk of diabetic ketoacidosis (DKA), a preventable and life-threatening condition, at diabetes onset in children during the pandemic.^4,17,18^

It is critical to know whether there was a sustained change in the incidence rates of both type 1 and type 2 diabetes in children because there are important implications for health resource planning for pediatric diabetes care, COVID-19–related and future pandemic-related public health measures, and immunization strategies. The primary objective of this systematic review and meta-analysis was to investigate whether there was a change in the incidence rate of types 1 and 2 diabetes in children and adolescents during the COVID-19 pandemic compared with before the pandemic. The secondary objective was to assess whether there was a change in the incidence rate of DKA among youths with new-onset diabetes during the COVID-19 pandemic.

## Methods

We prospectively registered this systematic review and meta-analysis on the PROSPERO database. The study followed the Meta-analysis of Observational Studies in Epidemiology (MOOSE) reporting guideline.^19^

### Data Sources and Search Strategy

We searched Medline (all segments), Embase, the Cochrane database, Scopus, and Web of Science for studies published from January 1, 2020, to March 28, 2023, in English. Our search strategy included subject headings and text word terms for *COVID-19* and (*diabetes type 1* or *2* or *diabetic ketoacidosis*) and *incidence* (eTable 1 in Supplement 1). We also conducted a gray literature search to identify studies published on government websites by searching for a combination of *COVID* and *diabetes* and statistical terms. We hand-searched the reference lists of all included studies and relevant systematic reviews.

### Eligibility Criteria

Studies were included if they (1) reported the number of incident cases of type 1 or 2 diabetes during the COVID-19 pandemic and before the pandemic in children and adolescents younger than 19 years, (2) had a minimum study period of 12 months prior to and during the COVID-19 pandemic, and (3) were published in English. Two reviewers (D.D., J.E.) used Covidence software^20^ to determine study eligibility. Conflicts were resolved by consensus or, if needed, in discussion with a third reviewer (R.S.). Interrater agreement at the screening and full-text stages was 95% and 90%, respectively.

### Data Extraction

We extracted the number of incident types 1 and 2 diabetes cases, study population size, and incidence rates of types 1 and 2 diabetes and DKA at diabetes diagnosis in the prepandemic and pandemic periods. The start of the pandemic period was defined according to the definition in each study. Two independent reviewers (D.D., J.E) extracted the data. Conflicts were resolved by consensus. Intercoder agreement was greater than 95%.

### Risk of Bias

We used the Risk of Bias in Non-randomized Studies of Exposure^21^ tool to assess the risk of bias in 7 domains (eTable 2 in Supplement 1). Two independent reviewers (D.D., J.E.) assessed the risk of bias for each of the included studies; conflicts were resolved by consensus or by a third reviewer (R.S).

### Statistical Analysis

We included studies in the meta-analysis if they reported the number of incident diabetes cases and the size of the study population for a minimum 12-month prepandemic period and a 12-month pandemic period. If those data were not reported, we contacted the corresponding author, requesting for them to share the data. If the study did not report the denominator (ie, study population) and we were unable to obtain it from the corresponding author, we included the study in a descriptive summary but excluded it from the meta-analysis because studies with missing denominators are likely to be of lower quality and, therefore, are not missing at random.^22^ Also, we wanted to focus the meta-analysis on the highest-quality studies. Studies with both pediatric and adult participants but no subgroup analysis for individuals younger than 19 years were included in the descriptive summary.

The number of incident cases and the size of the study population during the 12 months preceding the start of the pandemic period and the first 12 months following the start of the pandemic period were used to calculate the incidence rate ratio (IRR), the pooled IRR, and the corresponding 95% CIs. We conducted a meta-analysis of IRRs using common and random-effects approaches. Statistical heterogeneity was measured using the *I*^2^ statistic, and we assessed the statistical significance of between-study variation using a 2-sided *P* value of <.05.

Although some studies reported diabetes incidence for longer than 12 months in the prepandemic period, we included only data from 12 months preceding the start of the pandemic period in the meta-analysis because prepandemic diabetes incidence is known to have followed a seasonal pattern.^23,24^ Studies that had pandemic periods longer than 12 months are described in the narrative summary. Because seasonality changed during the pandemic,^23,24^ we conducted a post hoc additional analysis including only studies that reported more than 12 months of pandemic data, in which we compared incidence in the 12 months before the pandemic vs the first 12 months of the pandemic vs the second 12 months of the pandemic or the end of follow-up, whichever came first. We used the meta package in R, version 4.2.2 (R Project for Statistical Computing) for data analysis.^25,26^

## Results

**Figure 1.**
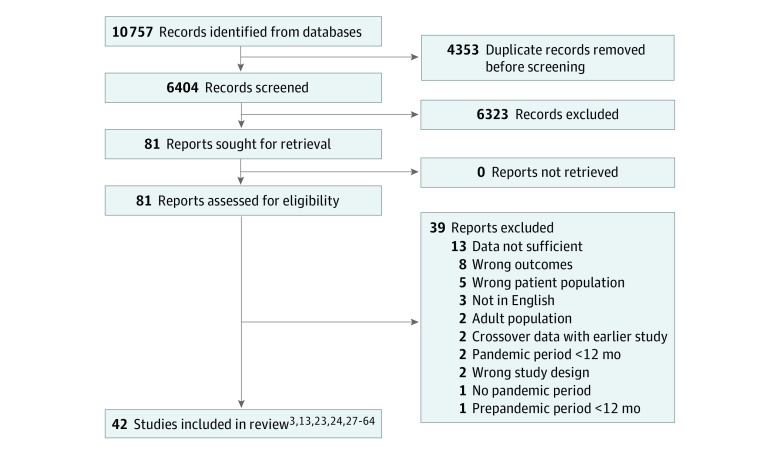
Flow Diagram of Study Selection

We identified 10 757 records, of which 4353 were duplicates (Figure 1). After the abstract review, we retrieved 81 full-text articles to determine eligibility. Forty-two records met the full inclusion criteria.^3,13,23,24,27,28,29,30,31,32,33,34,35,36,37,38,39,40,41,42,43,44,45,46,47,48,49,50,51,52,53,54,55,56,57,58,59,60,61,62,63,64^ The manual search of the included studies’ reference lists did not yield additional studies.

### Study Characteristics

Among the 42 included studies, there were 102 984 incident diabetes cases across both the prepandemic and the pandemic periods (Table 1). Twenty-four studies (57.1%) reported DKA incidence at diagnosis.^3,5,6,8,9,12,14,18,19,22,25,27,28,29,30,31,34,35,37,38,40,42,48,49^ Incident cases of type 1 and type 2 diabetes were reported in 36 studies (85.7%)^12,23,24,27,28,29,30,31,32,33,35,36,37,38,39,40,41,42,44,45,46,47,48,49,50,51,52,53,54,55,56,58,59,60,61,63^ and 9 studies (21.4%),^3,33,34,39,43,45,46,55,62^ respectively. Two studies (4.8%) did not distinguish between diabetes types.^13,57^ Thirty-two studies (76.2%) included children only,^3,12,13,23,24,27,28,29,30,31,32,35,36,37,38,39,40,41,42,44,48,49,50,51,52,53,56,57,58,60,61,63^ while the rest (10 [23.8%]) included both children and adults.^33,34,43,45,46,47,54,55,59,62^ Twenty-one studies (50.0%) were from Europe,^12,23,30,31,32,35,41,42,44,47,48,49,50,51,52,53,56,58,59,61,63^ 12 (28.6%) from North America,^3,13,33,34,38,39,43,45,46,54,57,62^ 7 (16.7%) from Asia,^27,28,29,36,37,40,60^ and 1 (2.4%) from Australia^55^; 1 study (2.4%)^24^ included data from multiple countries across different continents. Nine studies (21.4%) reported either the race or ethnicity of the study population,^3,34,39,43,45,46,47,54,62^ and 1 study reported socioeconomic status.^29^ All included studies were assessed to have an overall risk of bias rating of “some” (eTable 3 in Supplement 1).

**Table 1.** Study Characteristics

Source	Country	Study type and setting	Type of diabetes	Prepandemic period				Pandemic period			
				Age, mean, y	Female, %	Duration, mo (period)	Sample size	Age, mean, y	Female, %	Duration, mo (period)	Sample size
Alexandre et al,^30^ 2021	Portugal	Retrospective cohort, single site	1	10.0	55	12 (Apr 2019 to Mar 2020)	NR	12.3	37.0	12 (Apr 2020 to Mar 2021)	NR
Boboc et al,^31^2021	Romania	Retrospective cohort, single site	1	7.0	45.5	24 (Mar 2018 to Feb 2020)	973 750	7.2	49.0	12 (Mar 2020 to Feb 2021)	973 750
Dilek et al,^36^ 2021	Turkey	Retrospective cohort, single site	1	10.5	54.3	12 (Mar 2019 to Mar 2020)	NR	10.0	52.7	12 (Mar 2020 to Mar 2021)	NR
Kostopoulou et al,^41^ 2021	Greece	Prospective during pandemic with retrospective prepandemic, multisite	1	9.4	29.4	12 (Mar 2019 to Feb 2020)	NR	8.0	57.1	12 (Mar 2020 to Feb 2021)	NR
Mameli et al,^44^ 2021	Italy	Retrospective cohort, population based	1	2017, 8.7; 2018, 8.7; 2019, 8.9	2017, 45.0; 2018, 46.1; 2019, 49.4	36 (Jan 2017 to Dec 2019)	NR	8.5	43.0	12 (Jan to 2020)	
Marks et al,^45^ 2021	US	Retrospective cohort, single site	1 and 2	Type 1: 1 y PP, 10.0; 2 y PP, 10.0; T2D: 1 y PP, 14.1; 2 y PP, 13.8	1 y PP, 48.1; 2 y PP, 46.7 for both types	24 (Mar 2018 to Mar 2020)	NR	T1D, 10.0; T2D, 14.5	44.5 (Types 1 and 2)	12 (Mar 2020 to Mar 2021)	
Mohamed Haniffa et al,^47^ 2021	UK	Retrospective cross-sectional, single site	1	PP, 9.8	NR	12 (Apr 2019 to Mar 2020)	NR	8.5	NR	12 (Apr 2020 to Mar 2021)	NR
Moon et al,^48^ 2021	England	Retrospective cross -sectional, single site	1	NR	NR	48 (Mar 2016 to Mar 2020)	NR	NR	NR	12 (Mar 2020 to Mar 2021)	NR
Vlad et al,^52^ 2021	Romania	Retrospective cohort, population based	1	All pediatric age	NR	60 (Jan 2015 to Dec 2019)	3 057 024-3 318 667	All pediatric age	NR	12 (Jan to Dec 2020)	3 214 123-3 224 829
Al-Abdulrazzaq et al,^27^ 2022	Kuwait	Retrospective cross-sectional, population based	1	8.0	50.5	12 (Feb 2019 to Feb 2020)	805 851	8.2	73.4	12 (Feb 2020 to Feb 2021)	805 970
Al-Qahtani et al,^28^ 2022^a^	Saudi Arabia	Retrospective cohort, single site	1	NR	55.0 (All periods)	36 (Jan 2017 to Dec 2019)	NR	NR	55.0 (All periods)	24 (Jan 2020 to Dec 2021)	NR
Alassaf et al,^29^ 2022	Jordan	Retrospective cross-sectional, single site	1	Pediatric age	51.9	12 (Mar 2019 to Mar 2020)	NR	Pediatric age	55.4	12 (Mar 2020 to Mar 2021)	NR
Ansar et al,^57^ 2022	US	Retrospective cohort, single site	1 and 2	Pediatric age; most 6-16	NR	24 (Mar 2018 to Feb 2020)	NR	Pediatric age, most 6-16	NR	22 (Mar 2020 to Dec 2021)	NR
Australian Institute of Health and Welfare,^55^ 2022	Australia	Retrospective database, population based	1 and 2	Age for T1D, 0-19, and for T2D, 10-39^b^	NR for T1D	60 (Jan 2015 to Dec 2019)	T1D, 1 469 856-4 742 627; T2D, 27 210-28 517	Age for T1D, 0-19, and for T2D, 10-39^b^	NR	12 (Jan to Dec 2020)	T1D, 6 266 670; T2D, 26 881
Caetano et al,^56^ 2022^c^	Portugal	Retrospective cohort, single site	1	10.7	47.7	36 (Mar 2017 to Mar 2020)	NR	9.0	46.9	12 (Mar 2020 to Mar 2021)	NR
Cinek et al,^32^ 2022	Czechia	Retrospective cross-sectional, population based	1	NR	NR	60 (Jan 2015 to Dec 2019)	1 623 716-1 710 202	NR	NR	21 (Apr 2020 to Dec 2021)	1 718 145-1 719 741
Citron et al,^33^ 2022	US	Retrospective cross-sectional, single site	1 and 2	NR	NR	12 (Jan to Dec 2019)	NR	NR	NR	22 (Jan 2020 to Oct 2021)	NR
DeLacey et al,^34^ 2022	US	Retrospective cross-sectional, single site	2	14.1	54.2	60; (May 2015 to Apr 2020)	NR	14.1	45.9	12 (May 2020 to Apr 2021)	NR
Donbaloğlu et al,^37^ 2022	Turkey	Retrospective cohort, single site	1	NR	NR	24 (Apr 2018 to Apr 2020)	NR	9.4	71.0	12 (Apr 2020 to Apr 2021)	NR
Kamrath et al,^12^ 2022^d^	Germany	Retrospective cohort, population based	1	9.8	44.2	60 (Jan 2015 to Dec 2019)	NR	9.7	45.0	18 (Jan 2020 to Jun 2021)	NR
Gottesman et al,^38^ 2022	US	Retrospective cross-sectional, single site	1	9.7	NR	60 (Mar 2015 to Mar 2020)	1 405 967-1 410 787	9.6	56.7	12 (Mar 2020 to Mar 2021)	1 406 430
Guo et al,^39^ 2022	US	Retrospective cohort, population based	1 and 2	T1D, 11.0; T2D, 12.5	T1D, 48.7; T2D, 60.3	36 (Apr 2017 to Mar 2020)	NR	T1D, 11.0; T2D, 12.5	T1D, 48.7; T2D, 60.3	12 (Apr 2020 to Mar 2021)	NR
Kaya et al,^40^ 2022	Turkey	Retrospective cohort, single site	1	8.1	46.8	36 (Feb 2017 to Jan 2020)	NR	8.5	45.5	12 (Feb 2020 to Jan 2021)	NR
Leiva-Gea et al,^42^ 2022	Spain	Retrospective cross-sectional, multisite	1	NR	NR	60 (Jan 2015 to Dec 2019)	NR	NR	NR	15 (Jan 2020 to Mar 2021)	NR
Magge et al,^43^ 2022	US	Retrospective cross-sectional, multisite	2	14.4	55.0	24 (Mar 2018 to Feb 2020)	NR	14.4	45.0	12 (Mar 2020 to Feb 2021)	NR
Messaaoui et al,^61^ 2022	Belgium	Retrospective cohort, population based	1	Pediatric age	38	22 (Mar 2018 to Dec 2019)	NR	Pediatric age	56	22 (Mar 2020 to Dec 2021)	NR
Modarelli et al,^46^ 2022	US	Retrospective cohort, single site	1 and 2	T1D: 2 y PP, 9.9; 1 y PP, 10.4; T2D: 2 y PP, 9.9; 1 y PP, 9.9 y	T1D: 2 y PP, 55.0; 1 y PP, 55.0; T2D: 2 y PP, 59.0; 1 y PP, 56.0	24 (Apr 2018 to Mar 2020)	NR	T1D, 10.5; T2D, 9.9	T1D, 35.0; T2D, 53.0	12 (Apr 2020 to Mar 2021)	NR
Passanisi et al,^49^ 2022	Italy	Retrospective cross-section, population based	1	Pediatric age only	55.2 (All periods)	12 (Jan to Dec 2019)	252 792	Pediatric age in all periods	55.2 (All periods)	24 (Jan 2020 to Dec 2021)	245 602-247 723
Pietrzak et al,^50^ 2022	Poland	Retrospective cross-sectional, multisite	1	9.5	46.7 (All periods)	12 (Mar 2019 to Mar 2020)	6 454 756	9.5 (All periods)	46.7 (All periods)	12 (Mar 2020 to Mar 2021)	6 451 737
Raicevic et al,^51^ 2022	Montenegro	Retrospective cohort, population based	1	8.4	45.5 (All periods)	48 (Jan 2016 to Dec 2019)	111 475-113 302	8.4 (All periods)	45.5 (All periods)	12 (Jan 2020 to Dec 2020)	111 167
Reschke et al,^24^ 2022	Global	Retrospective cohort, multisite	1	1 y PP, 10.8; 2 y PP, 11.3	1 y PP, 46.2; 2 y PP, 47.6	24 (Jan 2018 to Dec 2019)	NR	Year 1, 10.6; year 2, 10.1	Year 1, 47.2; year 2, 44.8	24 (Jan 2020 to Dec 2021)	NR
Schiaffini et al,^35^ 2022	Italy	Retrospective cross-sectional, single site	1	NR	NR	36 (Jan 2017 to Dec 2019)	NR	NR	NR	24 (Jan 2020 to Dec 2021)	NR
Schmitt et al,^3^ 2022	US	Retrospective cross-sectional, single site	2	13.3	59.8 (All periods)	36 (Apr 2017 to Mar 2020)	NR	13.3 (All periods)	59.8 (All periods)	12 (Apr 2020 to Mar 2021)	NR
Shulman et al,^13^ 2022^e^	Canada	Retrospective cross-sectional, population based	1 and 2	9.2	48.7 (All periods)	36 (Jan 2017 to Dec 2019)	2 913 386-2 934 363	9.2 (All periods)	48.7 (All periods)	19 (Mar 2020 to Sep 2021)	2 700 178
van den Boom et al,^63^ 2022	Germany	Retrospective cohort, population based	1	All pediatric ages	NR	48 (Jan 2016 to Dec 2019)	15 221 437-15 330 502	All pediatric age	NR	24 (Jan 2020 to Dec 2021)	15 334 574-15 433 915
Vorgučin et al,^53^ 2022^f^	Serbia	Retrospective cohort, multisite	1	Pediatric age only	46.8 (All periods)	36 (Jan 2017 to Dec 2019)	NR	Pediatric age across all periods	46.8 (All periods)	24 (Jan 2020 to Dec 2021)	NR
Wolf et al,^54^ 2022	US	Retrospective cohort, multisite	1	10.6	48.7	12; (Jan to Dec 2019)	NR	10.2	46.3	12 (Jan to Dec 2020)	NR
Baechle et al,^23^ 2023	Germany	Retrospective cohort, population based	1	9.8	44.2	60 (Jan 2015 to Dec 2019)	NR	9.7	45.0	18 (Jan 2020 to Jun 2021)	NR
Gesuita et al,^58^ 2023	Italy	Retrospective cohort, population based	1	All pediatric age	NR	372 (Jan 2015 to Dec 2019)	718 593-762 431	All pediatric age	NR	24 (Jan 2020 to Dec 2021)	692 884-703 704
Giorda et al,^59^ 2023	Italy	Retrospective cohort, multisite	1	14.6	39.9	36 (Jan 2017 to Dec 2019)	18 049-18 685	14.6	39.9	24 (Jan 2020 to Dec 2021)	19 031-19 309
Matsuda et al,^60^ 2023	Japan	Retrospective cohort, population based	1	All pediatric ages	48.9	252; (Jan 1999 to Dec 2019)	136 123	All pediatric age	48.9	24 (Jan 2020 to Dec 2021)	136 123
Sasidharan Pillai et al,^62^ 2023	US	Retrospective cohort, single site	2	14.8	53.6	36 (Mar 2017 to Feb 2020)	NR	14.1	56.8	22 (Mar 2020 to Dec 2021)	NR

Abbreviations: NR, not reported; PP, prepandemic; T1D, type 1 diabetes; T2D, type 2 diabetes.

^a^ The denominator to calculate the incidence rate was the number of children presenting to the emergency department in each period.

^b^ The data for T2D were only included in the systematic review and were not meta-analyzed due to the inclusion of pediatric and adult persons.

^c^ This study contains duplicate data from a previous 2021 article published by the same authors. Only the more recent 2022 article was included in this systematic review.

^d^ This study was included in the systematic review but not the meta-analysis, as it contains partial duplicate data from the study by Baechle et al.^23^

^e^ Approximately 95% of all diabetes cases were T1D in this population.

^f^ Census data from 2011 were used to identify the at-risk pediatric population during both the prepandemic and the pandemic periods.

### Type 1 Diabetes Incidence Rate and Meta-Analysis

**Figure 2.**
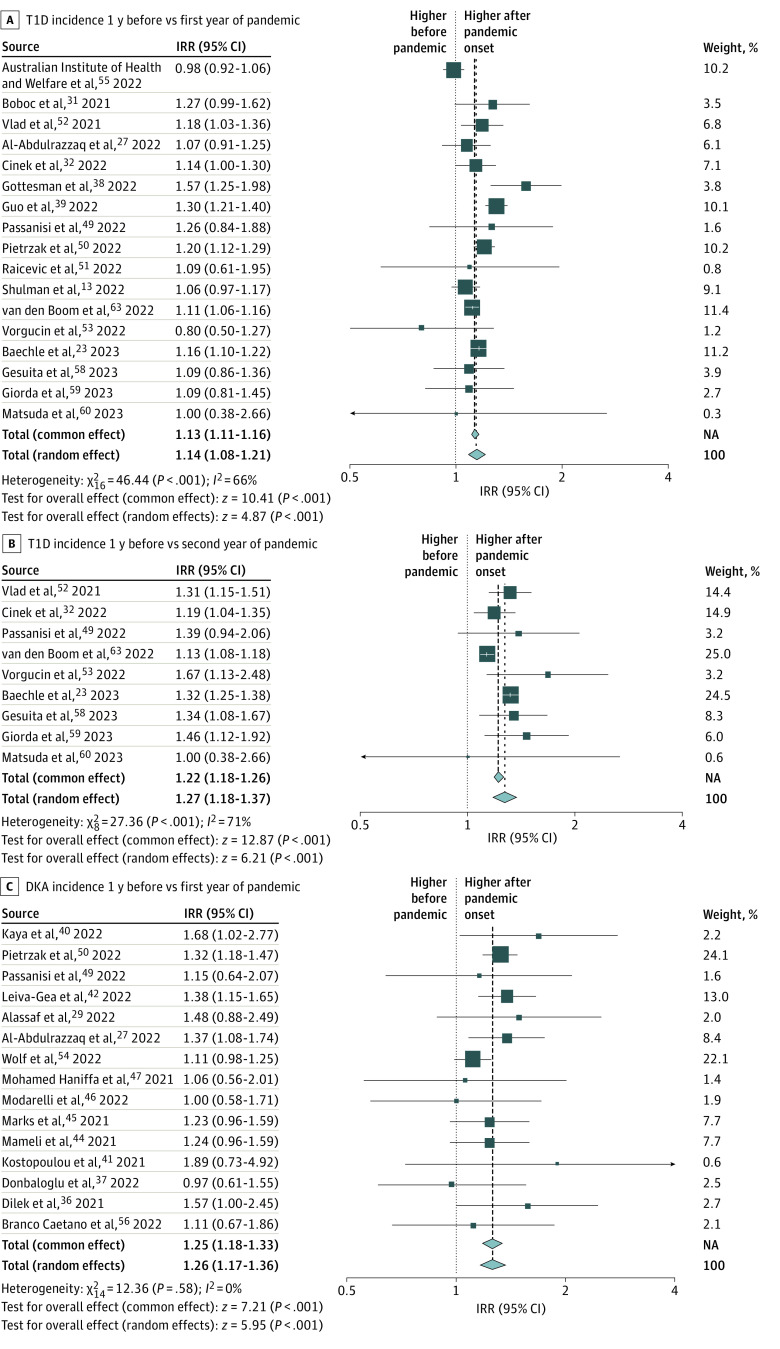
Forest Plots of Incidence Rate Ratios (IRRs) Squares indicate IRRs, with horizontal lines indicating 95% CIs and the size of the squares representing weight; diamonds indicate pooled estimates, with outer points of the diamonds indicating 95% CIs. DKA indicates diabetic ketoacidosis; NA, not applicable; T1D, type 1 diabetes.

In a random-effects meta-analysis of pooled data from 17 studies (40.5%) including 38 149 children and adolescents with newly diagnosed type 1 diabetes, there was a higher incidence rate of type 1 diabetes during the first year of the pandemic period compared with the prepandemic period (IRR, 1.14; 95% CI, 1.08-1.21) (Figure 2A).^13,23,24,27,31,32,38,39,49,50,51,53,55,58,59,60,63^ We excluded 2 studies^12,65^ from the meta-analysis because they contained overlapping data with more recent studies included in the meta-analysis. The data used to calculate the IRRs are available in Table 2. The unadjusted pooled IRR comparing the first year of the pandemic with the prepandemic period was 1.13 (95% CI, 1.11-1.16). Between-study heterogeneity was moderate (*I*^2^ = 66%).^22^ In our post hoc additional analysis, among studies that reported more than 12 months after pandemic onset, there was an increased incidence of diabetes during months 13 to 24 of the pandemic compared with the prepandemic period (IRR, 1.27; 95% CI, 1.18-1.37) (Figure 2B).^23,32,49,52,53,58,59,60,63^ The results of the remaining 20 studies, which reported the number of incident type 1 diabetes cases but were not included in the meta-analysis because they did not report the size of the study population, are summarized in Table 3.^24,28,29,30,33,35,36,37,40,41,42,44,45,46,47,48,54,57,61,64^ Of these, 15 (75.0%) reported an increase in the number of incident cases of type 1 diabetes during the first 12 months of the pandemic compared with during the 12 months before the pandemic.^33,35,36,37,40,41,42,44,45,46,47,48,54,61,64^

**Table 2.** Incident Cases of Pediatric Diabetes and DKA and IRRs for Studies Included in the Meta-Analyses

Source	1 y Prepandemic			First-year postpandemic start				Second-year postpandemic start				
	Incident diabetes cases, No.	Population	Incidence rate, per 100 000 individuals	Incident diabetes cases, No.	Population	Incidence rate, per 100 000 individuals	IRR vs prepandemic	Months, No.	Incident diabetes cases, No.	Population	Incidence rate, per 100 000 individuals	IRR vs prepandemic
**Diabetes incidence meta-analysis**												
Boboc et al,^31^ 2021	116	973 750	11.91	147	973 750	15.10	1.27	NR	NR	NR	NR	NR
Vlad et al,^52^ 2021	367	3 231 435	11.36	433	3 224 829	13.43	1.18	12	480	3 214 123	14.93	1.31
Al-Abdulrazzaq et al,^27^ 2022	303	805 851	37.60	324	805 970	40.20	1.07	NR	NR	NR	NR	NR
Australian Institute of Health and Welfare,^55^ 2022	1510	6 280 835	24.04	1482	6 266 670	23.65	0.98	NR	NR	NR	NR	NR
Cinek et al,^32^ 2022	409	1 710 202	23.92	468	1 719 741	27.21	1.14	12	488	1 718 145	28.40	1.19
Gottesman et al,^38^ 2022	119	1 406 353	8.46	187	1 406 430	13.30	1.57	NR	NR	NR	NR	NR
Guo et al,^39^ 2022	1283	4 953 668	25.90	1541	4 572 700	33.70	1.30	NR	NR	NR	NR	NR
Passanisi et al,^49^ 2022	43	252 792	17.01	53	247 723	21.39	1.26	12	58	245 602	23.62	1.39
Pietrzak et al,^50^ 2022	1391	6 454 756	21.55	1671	6 451 737	25.90	1.20	NR	NR	NR	NR	NR
Raicevic et al,^51^ 2022	22	111 475	19.74	24	111 167	21.59	1.09	NR	NR	NR	NR	NR
Shulman et al,^13^ 2022	888	2 913 386	30.48	874	2 700 178	32.37	1.06	7	696	2 700 178	32.22	1.06
van den Boom et al,^63^ 2022	3646	15 330 502	23.78	4046	15 334 574	26.38	1.11	12	4153	15 433 915	26.91	1.13
Vorgučin et al,^53^ 2022	40	387 302	10.33	32	387 302	8.26	0.80	12	67	387 302	17.30	1.67
Baechle et al,^23^ 2023	2903	14 160 976	20.50	3338	14 084 388	23.70	1.16	12	3706	13 726 908	27.00	1.32
Gesuita et al,^58^ 2023	143	718 593	19.90	152	703 704	21.60	1.09	12	185	692 884	26.70	1.34
Giorda et al,^59^ 2023	89	535 177	16.63	95	525 442	18.08	1.05	12	126	517 879	24.33	1.46
Matsuda et al,^60^ 2023	8	136 123	5.88	8	136 123	5.88	1.00	12	8	136 123	5.88	1.00
Pooled^a^	13 280	60 363 176	22.00	14 875	59 652 428	24.94	NA	NA	9271	36 072 881	25.70	NA
**DKA incidence meta-analysis**												
Caetano et al,^56^ 2021	64	165	38 787.88	19	44	43 181.82	1.11	NR	NR	NR	NR	NR
Dilek et al,^36^ 2021	27	46	58 695.65	68	74	91 891.89	1.57	NR	NR	NR	NR	NR
Kostopoulou et al,^41^ 2021	6	17	35 294.12	14	21	66 666.67	1.89	NR	NR	NR	NR	NR
Mameli et al,^44^ 2021	184	502	36 653.39	91	201	45 273.63	1.24	NR	NR	NR	NR	NR
Marks et al,^45^ 2021	145	310	46 774.19	105	182	57 692.31	1.23	NR	NR	NR	NR	NR
Mohamed Haniffa et al,^47^ 2021	14	28	50 000.00	28	53	52 830.19	1.06	NR	NR	NR	NR	NR
Al-Abdulrazzaq et al,^27^ 2021	113	303	37 293.73	166	324	51 234.57	1.37	NR	NR	NR	NR	NR
Alassaf et al,^29^ 2021	29	83	34 939.76	28	54	51 851.85	1.48	NR	NR	NR	NR	NR
Donbaloğlu et al,^37^ 2022	43	78	55 128.21	30	56	53 571.43	0.97	NR	NR	NR	NR	NR
Kaya et al,^40^ 2022	32	79	40 506.33	30	44	68 181.82	1.68	NR	NR	NR	NR	NR
Leiva-Gea et al,^42^ 2022	377	1085	34 746.54	172	359	47 910.86	1.38	NR	NR	NR	NR	NR
Modarelli et al,^46^ 2022	31	62	50 000.00	23	46	50 000.00	1.00	NR	NR	NR	NR	NR
Passanisi et al,^49^ 2022	19	43	44 186.05	27	53	46 846.85	1.06	12	25	58	43 103.45	0.98
Pietrzak et al,^50^ 2022	521	1391	37 455.07	826	1671	49 431.48	1.32	NR	NR	NR	NR	NR
Wolf et al,^54^ 2022	493	1277	38 606.11	599	1399	42 816.30	1.11	NR	NR	NR	NR	NR
Pooled	2098	5469	38 361.68	2226	4581	48 592.01	1.27	NR	NR	NR	NR	NR

Abbreviations: DKA, diabetic ketoacidosis; IRR, incident rate ratio; NR, not reported.

^a^ Pooled data for the second year of the postpandemic period exclude the data from Shulman et al^13^ due to insufficient duration of observation.

**Table 3.** Incident Types 1 and 2 Diabetes Cases Before and During the COVID-19 Pandemic Reported in Studies Not Included in the Meta-Analysis

Source	Prepandemic period		Pandemic period	
	Duration, mo	Incident cases, No.	Duration, mo	Incident cases, No.
**Type 1 diabetes**				
Alexandre et al,^30^ 2021	12	27	12	20
Al-Qahtani et al,^28^ 2022	12	260	12	167
Dilek et al,^36^ 2021	12	46	12	74
Kostopoulou et al,^41^ 2021	12	17	12	21
Mameli et al,^44^ 2021	36	624	12	256
Marks et al,^45^ 2021	24	310	12	182
Mohamed Haniffa et al,^47^ 2021	12	28	12	53
Moon et al,^48^ 2021	48	19-28^a^	12	30
Alassaf et al,^29^ 2022	12	83	12	54
Ansar et al,^57^ 2022^a^	24	NR	22	NR
Citron et al,^33^ 2022	12	35	20	82
Donbaloğlu et al,^37^ 2022	24	78	12	56
Kaya et al,^40^ 2022	36	79	12	44
Leiva-Gea et al,^42^ 2022	60	1085	15	359
Messaaoui et al,^61^ 2022	22	87	22	147
Modarelli et al,^46^ 2022	24	62	12	46
Reschke et al,^24^ 2022	24	9090	24	8190
Schiaffini et al,^35^ 2022	36	290	24	220
Wolf et al,^54^ 2022	12	1277	12	1399
Knip et al,^64^ 2023	54	2096	18	785
**Type 2 diabetes**				
Marks et al,^45^ 2021	24	104	12	141
Ansar et al,^57^ 2022^b^	24	NR	22	NR
Australian Institute of Health and Welfare,^55^ 2022	60	9330	12	1540
Citron et al,^33^ 2022	12	8	22	43
DeLacey et al,^34^ 2022	60	271	12	159
Guo et al,^39^ 2022	36	1852	12	701
Magge et al,^43^ 2022	24	1651	12	1463
Modarelli et al,^46^ 2022	24	33	12	53
Schmitt et al,^3^ 2022	36	400	12	232
Sasidharan Pillai et al,^62^ 2023	38	56	22	88

Abbreviation: NR, not reported.

^a^ Per year.

^b^ This study did not differentiate between types 1 and 2 diabetes cases when reporting incidence data.

### Type 2 Diabetes

Ten of 42 studies (23.8%) reported the number of incident type 2 diabetes cases^3,33,34,39,43,45,46,55,57,62^; however, only 1 of those (10.0%) reported the size of the study populations.^55^ Therefore, we were unable to conduct a meta-analysis comparing the incidence rate of type 2 diabetes between periods. We summarize the results of these studies in Table 3. Eight studies (80.0%) reported an increase in the number of incident cases of type 2 diabetes during the first 12 months of the pandemic compared with during the 12 months before the pandemic.^3,33,34,39,43,45,46,62^

### DKA Incidence Rate Meta-Analysis

In a random-effects meta-analysis of pooled data from 15 studies (35.7%) including a total of 4324 children and adolescents with DKA, the incidence rate of DKA was higher during the pandemic period compared with the prepandemic period (IRR, 1.26; 95% CI, 1.17-1.36) (Figure 2C).^27,29,36,37,40,41,42,44,45,46,47,49,50,54,65^ Between-study heterogeneity was minimal (*I*^2^ = 0%).

## Discussion

In this systematic review and meta-analysis, in 17 studies including 38 149 children and adolescents with newly diagnosed type 1 diabetes,^13,23,24,27,31,32,38,39,49,50,51,53,55,58,59,60,63^ we found that the incidence rate of type 1 diabetes was 1.14 times higher in the first year and 1.27 times higher in the second year after the onset of the COVID-19 pandemic compared with before the pandemic. In 15 studies including a total of 4324 children and adolescents with DKA,^27,29,36,37,40,41,42,44,45,46,47,49,50,54,65^ we also found that the incidence rate of DKA at diagnosis was 1.26 times higher in the first year after the onset of the COVID-19 pandemic compared with before the pandemic. The magnitude of increase in the incidence rate of type 1 diabetes that we observed after the onset of the pandemic was greater than the expected 3% to 4% annual increase in the incidence rate based on prepandemic temporal trends in Europe.^9^

Our findings are similar to those of another recent meta-analysis by Rahmati et al^4^ that examined the incidence rate of type 1 diabetes and ketoacidosis in children during the COVID-19 pandemic in 2020 and during the same period in 2019. We compared the rate ratios reported in that meta-analysis by the length of their pandemic observation period. We found that studies with a pandemic period of 6 months or less had a lower estimated incidence rate compared with studies with a pandemic period of 12 months or greater (eFigure in Supplement 1). Our systematic review adds important new information because it included studies that examined the incidence of both types 1 and 2 diabetes in children and adolescents, included additional data from later in the pandemic, and required at least 12 months of observation in both the pandemic and the prepandemic periods to account for the prepandemic seasonality of diabetes incidence and changes in seasonality during the pandemic that differed between Europe and North America.^23,24^

We found substantial heterogeneity in the meta-analysis of diabetes incidence but not in the meta-analysis of DKA incidence. It is presumptive to assume why this occurred; however, some potential explanations include that higher within-study variation in the DKA meta-analysis may have resulted in a lower *I*^2^ value,^66^ and other demographic, geographical, and methodologic factors may have led to increased heterogeneity between studies in the diabetes incidence meta-analysis.

Purported direct mechanisms to explain the association between new-onset diabetes and prior SARS-CoV-2 infection include evidence that the SARS-CoV-2 entry receptor ACE2 is expressed on insulin-producing β cells, SARS-CoV-2 infection contributes to dysregulation of glucose metabolism, and individuals who have an increased susceptibility to diabetes are especially vulnerable following SARS-CoV-2 infection because dysregulated glucose metabolism and direct viral damage to β cells impairs their compensatory mechanisms, leading to β-cell exhaustion.^7^ However, there is no clear underlying mechanism explaining the association between SARS-CoV-2 infection and subsequent increased risk of incident diabetes.^7,8^ While there are reports of an association between SARS-CoV-2 infection and subsequent increased risk of incident type 1 diabetes in children using routinely collected health record data,^5,6,67^ there are concerns about the validity of such studies because the data sets used did not capture asymptomatic SARS-CoV-2 infections in children. Population-based studies that reported an increased incidence rate of type 1 diabetes in children and adolescents during the pandemic did not find an increase in the frequency of autoantibody-negative type 1 diabetes^12,23,68^; this suggests that the increase in incidence may be due to an immune-mediated mechanism.

Proposed indirect effects of the COVID-19 pandemic and containment measures that may be associated with diabetes incidence include changes in lifestyle, change in the pattern of pediatric non–COVID-19 infections, and increased stress and social isolation.^12,69,70,71^ It has been proposed that frequent respiratory or enteric infections in children are potential triggers for islet autoimmunity, promote progression to overt type 1 diabetes, or are precipitating stressors.^72^ Pandemic containment measures were associated with a decrease in viral respiratory and gastrointestinal tract infections among children.^69^ Given this finding, the observed increased incidence rate of type 1 diabetes during the pandemic is contrary to what would be expected based on the decrease in viral infections among children during the pandemic.

There may have initially been a catch-up effect caused by lower incidence rates of pediatric diabetes early in the pandemic, possibly due to delays in diagnoses associated with hesitancy to seek care or barriers to access care.^12,13,14^ However, the reported incidence of diabetes remained increased in studies that included data from beyond the first year of the pandemic.^23,32,49,52,53,58,59,60,63^ Furthermore, there appears to have been a disruption to the historic seasonal pattern of autoantibody-positive diabetes incidence in children.^23,24^ The reasons for this remain uncertain but may be related to the effects of COVID-19 containment strategies, such as lockdowns, both at the beginning of the pandemic and at subsequent times in different countries.^73^

There are limited data about the change in the incidence rate of pediatric type 2 diabetes during the COVID-19 pandemic. The studies included in this systematic review and meta-analysis described an increase in the number of incident type 2 diabetes cases between periods but had insufficient data reported to assess whether there was also an increase in the incidence rate of childhood type 2 diabetes after the onset of the pandemic. Population-based studies that can measure the size of the study population (denominator) and therefore determine whether there has been a change in the incidence rate of type 2 diabetes in children and adolescents since the onset of the COVID-19 pandemic are needed.

We found an increased incidence rate of DKA at diabetes diagnosis among children and adolescents during the pandemic. This is concerning because DKA is preventable and an important cause of morbidity and mortality and is associated with long-term poor glycemic management.^74,75^ An international study that used data from 13 pediatric diabetes registries reported a prevalence of DKA at diagnosis in 2020 and 2021 that was higher than the predicted prevalence based on prepandemic years 2006 to 2019.^76^ A population-based study in Germany^77^ found that the regional incidence of COVID-19 cases and deaths was associated with an increased risk of DKA at diagnosis, suggesting that the local severity of the pandemic, rather than the pandemic containment measures, may have led to delayed health care use and diagnosis. In Ontario, Canada, there was a higher DKA rate among those who had no precedent primary care visits and a pattern of fewer emergency department visits during the pandemic,^14^ suggesting that delays in diagnosis of diabetes resulting in DKA may reflect hesitancy to seek care or barriers to access emergency care. Individuals living in areas with high COVID-19 positivity reported more hesitancy to seek emergency care for children.^78^ Therefore, hesitancy to seek care may be an important factor in the observed increased risk of DKA during the pandemic.

There is concern about widespread negative consequences of the COVID-19 pandemic for child and adolescent health inequities.^79^ However, relatively few studies examining changes in the incidence rate of pediatric diabetes since the onset of the COVID-19 pandemic have reported the socioeconomic status, race, or ethnicity of the study population. Such information would elucidate whether health disparities in the incidence rates of diabetes and DKA widened during the pandemic.^80,81^

### Implications

The results of our systematic review and meta-analysis demonstrated an increased incidence in childhood diabetes after the onset of the COVID-19 pandemic. The increased incidence rate of type 1 diabetes appeared to persist beyond the first year of the pandemic; this has important resource implications given the limited personnel resources in pediatric diabetes care to provide initial diabetes education at diagnosis and for long-term care. Future studies examining longer-term trends of incident types 1 and 2 diabetes may assess whether the increased incidence rate of type 1 diabetes continued and whether there was an increased incidence rate of pediatric type 2 diabetes. A better understanding of the possible direct effects of SARS-CoV-2 infection and the indirect effects of pandemic-related containment measures on incident diabetes in children is needed.

The increased prevalence of DKA at the time of diabetes diagnosis brings to light the need to identify the gaps in the pathway from the time when children develop signs of diabetes to subsequent diagnosis with DKA. This knowledge is needed to inform the development and implementation of effective strategies to prevent DKA at diagnosis in children. These may include public and health care professional–facing awareness campaigns and addressing hesitancy to seek emergency care.^78,82^

### Limitations

This study has limitations. Our search was restricted to studies published in English, and the included studies did not represent all regions of the world, limiting the generalizability of our findings worldwide. We included only studies that reported the incidence of DKA at diabetes diagnosis among studies that met our eligibility criteria, which required reporting incident diabetes cases in both study periods. Some studies included in our systematic review did not measure diabetes autoantibodies to confirm whether an individual had type 1 or another type of diabetes; thus, there may be a risk of misclassification of diabetes type.

## Conclusions

This systematic review and meta-analysis found increased incidence rates of type 1 diabetes and DKA in children and adolescents during vs before the COVID-19 pandemic. Our findings underscore the need to dedicate resources to supporting an acute increased need for pediatric and ultimately young adult diabetes care and strategies to prevent DKA in patients with new-onset diabetes. Although prospective data examining whether this trend has persisted are needed, our findings suggest the need to elucidate possible underlying direct and indirect mechanisms to explain this increase. Furthermore, there is a paucity of data about socioeconomic, racial, and ethnic disparities in the incidence rate of diabetes during the COVID-19 pandemic; this gap must be filled to inform equitable strategies for intervention.

## Notes

Supplement 1. **eTable 1.** Subject Database and Gray Literature Search Strategies **eTable 2.** Risk-of-Bias Evaluation Criteria Domains **eTable 3.** Risk-of-Bias Assessments for Included Studies, Using the ROBINS-E Tool **eFigure.** Rate Ratios Reported in the Meta-analysis by Rahmati et al, by Length of the Pandemic Observation Period

Supplement 2.
Data Sharing Statement

## References

[1] Gregory GA, Robinson TIG, Linklater SE, ; International Diabetes Federation Diabetes Atlas Type 1 Diabetes in Adults Special Interest Group. Global incidence, prevalence, and mortality of type 1 diabetes in 2021 with projection to 2040: a modelling study. Lancet Diabetes Endocrinol. 2022;10(10):741-760. doi:10.1016/S2213-8587(22)00218-2 36113507

[2] Lawrence JM, Divers J, Isom S, ; SEARCH for Diabetes in Youth Study Group. Trends in prevalence of type 1 and type 2 diabetes in children and adolescents in the US, 2001-2017. JAMA. 2021;326(8):717-727. doi:10.1001/jama.2021.11165 34427600 PMC8385600

[3] Schmitt JA, Ashraf AP, Becker DJ, Sen B. Changes in type 2 diabetes trends in children and adolescents during the COVID-19 pandemic. J Clin Endocrinol Metab. 2022;107(7):e2777-e2782. doi:10.1210/clinem/dgac209 35377436 PMC8992346

[4] Rahmati M, Keshvari M, Mirnasuri S, . The global impact of COVID-19 pandemic on the incidence of pediatric new-onset type 1 diabetes and ketoacidosis: a systematic review and meta-analysis. J Med Virol. 2022;94(11):5112-5127. doi:10.1002/jmv.27996 35831242 PMC9350204

[5] Barrett CE, Koyama AK, Alvarez P, . Risk for newly diagnosed diabetes >30 days after SARS-CoV-2 infection among persons aged <18 years—United States, March 1, 2020–June 28, 2021. MMWR Morb Mortal Wkly Rep. 2022;71(2):59-65. doi:10.15585/mmwr.mm7102e2 35025851 PMC8757617

[6] Kendall EK, Olaker VR, Kaelber DC, Xu R, Davis PB. Association of SARS-CoV-2 infection with new-onset type 1 diabetes among pediatric patients from 2020 to 2021. JAMA Netw Open. 2022;5(9):e2233014. doi:10.1001/jamanetworkopen.2022.33014 36149658 PMC9508649

[7] Groß R, Kleger A. COVID-19 and diabetes—where are we now? Nat Metab. 2022;4(12):1611-1613. doi:10.1038/s42255-022-00691-w 36369292

[8] Accili D. Can COVID-19 cause diabetes? Nat Metab. 2021;3(2):123-125. doi:10.1038/s42255-020-00339-7 33432203 PMC8892570

[9] Patterson CC, Harjutsalo V, Rosenbauer J, . Trends and cyclical variation in the incidence of childhood type 1 diabetes in 26 European centres in the 25 year period 1989-2013: a multicentre prospective registration study. Diabetologia. 2019;62(3):408-417. doi:10.1007/s00125-018-4763-3 30483858

[10] Gerasimidi Vazeou A, Kordonouri O, Witsch M, ; SWEET Study Group. Seasonality at the clinical onset of type 1 diabetes—lessons from the SWEET database. Pediatr Diabetes. 2016;17(suppl 23):32-37. doi:10.1111/pedi.12433 28334496

[11] Patterson CC, Gyürüs E, Rosenbauer J, . Seasonal variation in month of diagnosis in children with type 1 diabetes registered in 23 European centers during 1989-2008: little short-term influence of sunshine hours or average temperature. Pediatr Diabetes. 2015;16(8):573-580. doi:10.1111/pedi.12227 25316271

[12] Kamrath C, Rosenbauer J, Eckert AJ, . Incidence of type 1 diabetes in children and adolescents during the COVID-19 pandemic in Germany: results from the DPV Registry. Diabetes Care. 2022;45(8):1762-1771. doi:10.2337/dc21-0969 35043145

[13] Shulman R, Cohen E, Stukel TA, Diong C, Guttmann A. Examination of trends in diabetes incidence among children during the COVID-19 pandemic in Ontario, Canada, from March 2020 to September 2021. JAMA Netw Open. 2022;5(7):e2223394. doi:10.1001/jamanetworkopen.2022.23394 35877126 PMC9315418

[14] Shulman R, Nakhla M, Diong C, Stukel TA, Guttmann A. Health care use prior to diabetes diagnosis in children before and during COVID. Pediatrics. 2022;150(4):e2022058349. doi:10.1542/peds.2022-058349 35945681

[15] An R. Projecting the impact of the coronavirus disease-2019 pandemic on childhood obesity in the United States: a microsimulation model. J Sport Health Sci. 2020;9(4):302-312. doi:10.1016/j.jshs.2020.05.006 32454174 PMC7250129

[16] Hannon TS, Rao G, Arslanian SA. Childhood obesity and type 2 diabetes mellitus. Pediatrics. 2005;116(2):473-480. doi:10.1542/peds.2004-2536 16061606

[17] Elgenidy A, Awad AK, Saad K, . Incidence of diabetic ketoacidosis during COVID-19 pandemic: a meta-analysis of 124,597 children with diabetes. Pediatr Res. 2023;93(5):1149-1160. doi:10.1038/s41390-022-02241-2 35953513 PMC9366798

[18] Sellers EAC, Pacaud D. Diabetic ketoacidosis at presentation of type 1 diabetes in children in Canada during the COVID-19 pandemic. Paediatr Child Health. 2021;26(4):208-209. doi:10.1093/pch/pxab017 34127934 PMC8083512

[19] Brooke BS, Schwartz TA, Pawlik TM. MOOSE reporting guidelines for meta-analyses of observational studies. JAMA Surg. 2021;156(8):787-788. doi:10.1001/jamasurg.2021.0522 33825847

[20] Covidence Systematic Review Software. Veritas Health Innovation; 2022.

[21] ROBINS-E Development Group. Risk of Bias in Non-randomized Studies of Exposure. June 1, 2022. Accessed November 15, 2022. https://www.riskofbias.info/welcome/robins-e-tool

[22] Deeks JJ, Higgins JPT, Altman DG; Cochrane Statistical Methods Group. Analysing data and undertaking meta-analyses. In: Higgins J, Thomas J, eds. *Cochrane Handbook for Systematic Reviews of Interventions*. Version 6.3. Cochrane; 2022. Accessed November 15, 2022. http://www.training.cochrane.org/handbook

[23] Baechle C, Eckert A, Kamrath C, . Incidence and presentation of new-onset type 1 diabetes in children and adolescents from Germany during the COVID-19 pandemic 2020 and 2021: current data from the DPV registry. Diabetes Res Clin Pract. 2023;197:110559. doi:10.1016/j.diabres.2023.110559 36758641

[24] Reschke F, Lanzinger S, Herczeg V, ; SWEET Study Group. The COVID-19 pandemic affects seasonality, with increasing cases of new-onset type 1 diabetes in children, from the worldwide SWEET registry. Diabetes Care. 2022;45(11):2594-2601. doi:10.2337/dc22-0278 36166593

[25] R Core Team. *R: A Language and Environment for Statistical Computing*. Version 4.2.2. R Foundation for Statistical Computing; 2022.

[26] Balduzzi S, Rücker G, Schwarzer G. How to perform a meta-analysis with R: a practical tutorial. Evid Based Ment Health. 2019;22(4):153-160. doi:10.1136/ebmental-2019-300117 31563865 PMC10231495

[27] Al-Abdulrazzaq D, Alkandari A, Alhusaini F, ; CODeR group. Higher rates of diabetic ketoacidosis and admission to the paediatric intensive care unit among newly diagnosed children with type 1 diabetes in Kuwait during the COVID-19 pandemic. Diabetes Metab Res Rev. 2022;38(3):e3506. doi:10.1002/dmrr.3506 34679258 PMC8646429

[28] Al-Qahtani MH, Bukhamseen FM, Al-Qassab AT, . The impact of COVID-19 lockdown on the incidence of type 1 DM and the glycemic control of diabetic children: findings from a teaching hospital, Saudi Arabia. Rev Diabet Stud. 2022;18(3):152-156. doi:10.1900/RDS.2022.18.152 36309774 PMC9652708

[29] Alassaf A, Gharaibeh L, Ibrahim S, . Effect of COVID-19 pandemic on presentation and referral patterns of newly diagnosed children with type 1 diabetes in a developing country. J Pediatr Endocrinol Metab. 2022;35(7):859-866. doi:10.1515/jpem-2022-0136 35607289

[30] Alexandre MI, Henriques AR, Cavaco D, . New-onset type 1 diabetes in children and COVID-19. Acta Med Port. 2021;34(9):642-643. doi:10.20344/amp.16412 34321150

[31] Boboc AA, Novac CN, Ilie MT, . The impact of SARS-CoV-2 pandemic on the new cases of T1DM in children: a single-centre cohort study. J Pers Med. 2021;11(6):551. doi:10.3390/jpm11060551 34199272 PMC8231839

[32] Cinek O, Slavenko M, Pomahačová R, ; ČENDA Register. Type 1 diabetes incidence increased during the COVID-19 pandemic years 2020-2021 in Czechia: results from a large population-based pediatric register. Pediatr Diabetes. 2022;23(7):956-960. doi:10.1111/pedi.13405 35982508 PMC9538386

[33] Citron K, Stein CR, Ilkowitz J, Gonzalez JE, Joseph V, Gallagher MP. Presentation of new onset diabetes in youth during the COVID-19 pandemic. Diabetes. 2022;71(suppl1):168-LB.

[34] DeLacey S, Arzu J, Levin L, Ranganna A, Swamy A, Bianco ME. Impact of SARS-CoV2 on youth onset type 2 diabetes new diagnoses and severity. J Diabetes. 2022;14(8):532-540. doi:10.1111/1753-0407.13301 36040204 PMC9426273

[35] Schiaffini R, Deodati A, Rapini N, Pampanini V, Cianfarani S. Increased incidence of childhood type 1 diabetes during the COVID-19 pandemic: figures from an Italian tertiary care center. J Diabetes. 2022;14(8):562-563. doi:10.1111/1753-0407.13298 35916392 PMC9426276

[36] Dilek SÖ, Gürbüz F, Turan İ, Celiloğlu C, Yüksel B. Changes in the presentation of newly diagnosed type 1 diabetes in children during the COVID-19 pandemic in a tertiary center in Southern Turkey. J Pediatr Endocrinol Metab. 2021;34(10):1303-1309. doi:10.1515/jpem-2021-0287 34291625

[37] Donbaloğlu Z, Tuhan H, Tural Kara T, . The examination of the relationship between COVID-19 and new-onset type 1 diabetes mellitus in children. Turk Arch Pediatr. 2022;57(2):222-227. doi:10.5152/TurkArchPediatr.2022.21284 35383019 PMC9366160

[38] Gottesman BL, Yu J, Tanaka C, Longhurst CA, Kim JJ. Incidence of new-onset type 1 diabetes among US children during the COVID-19 global pandemic. JAMA Pediatr. 2022;176(4):414-415. doi:10.1001/jamapediatrics.2021.5801 35072727 PMC8787677

[39] Guo Y, Bian J, Chen A, . Incidence trends of new-onset diabetes in children and adolescents before and during the COVID-19 pandemic: findings from Florida. Diabetes. 2022;71(12):2702-2706. doi:10.2337/db22-0549 36094294 PMC9750945

[40] Kaya G, Cimbek EA, Yeşilbaş O, Bostan YE, Karagüzel G. A Long-term comparison of presenting characteristics of children with newly diagnosed type 1 diabetes before and during the COVID-19 pandemic. J Clin Res Pediatr Endocrinol. 2022;14(3):267-274. doi:10.4274/jcrpe.galenos.2022.2021-10-2 35308015 PMC9422920

[41] Kostopoulou E, Eliopoulou MI, Rojas Gil AP, Chrysis D. Impact of COVID-19 on new-onset type 1 diabetes mellitus—a one-year prospective study. Eur Rev Med Pharmacol Sci. 2021;25(19):5928-5935. 34661251 10.26355/eurrev_202110_26869

[42] Leiva-Gea I, Fernández CA, Cardona-Hernandez R, ; Diabetes Group of the Spanish Pediatric Endocrinology Society (SEEP). Increased presentation of diabetic ketoacidosis and changes in age and month of type 1 diabetes at onset during the COVID-19 pandemic in Spain. J Clin Med. 2022;11(15):4338. doi:10.3390/jcm11154338 35893428 PMC9369057

[43] Magge SN, Wolf RM, Pyle L, ; COVID-19 and Type 2 Diabetes Consortium. The coronavirus disease 2019 pandemic is associated with a substantial rise in frequency and severity of presentation of youth-onset type 2 diabetes. J Pediatr. 2022;251:51-59.e2. doi:10.1016/j.jpeds.2022.08.010 35985535 PMC9383958

[44] Mameli C, Scaramuzza A, Macedoni M, . Type 1 diabetes onset in Lombardy region, Italy, during the COVID-19 pandemic: the double-wave occurrence. EClinicalMedicine. 2021;39:101067. doi:10.1016/j.eclinm.2021.101067 34430836 PMC8365462

[45] Marks BE, Khilnani A, Meyers A, . Increase in the diagnosis and severity of presentation of pediatric type 1 and type 2 diabetes during the COVID-19 pandemic. Horm Res Paediatr. 2021;94(7-8):275-284. doi:10.1159/000519797 34564073 PMC8805060

[46] Modarelli R, Sarah S, Ramaker ME, Bolobiongo M, Benjamin R, Gumus Balikcioglu P. Pediatric diabetes on the rise: trends in incident diabetes during the COVID-19 pandemic. J Endocr Soc. 2022;6(4):bvac024. doi:10.1210/jendso/bvac024 35265783 PMC8900286

[47] Mohamed Haniffa F, Karthikeyan S, Dhanji A, Agwu JC. Impact of pandemic on profile and outcome of patients with new onset type 1 diabetes. Pediatr Diabetes. 2021;22(S30):41.

[48] Moon R, Van Boxel E, Berg E, Trevelyan N. Increased diabetic ketoacidosis at presentation of type 1 diabetes mellitus—a result of the COVID-19 pandemic or longer-term increasing trend? *Endocrine Abstracts*. 2021;78:P13.

[49] Passanisi S, Salzano G, Aloe M, . Increasing trend of type 1 diabetes incidence in the pediatric population of the Calabria region in 2019-2021. Ital J Pediatr. 2022;48(1):66. doi:10.1186/s13052-022-01264-z 35509062 PMC9066995

[50] Pietrzak I, Michalak A, Seget S, . Diabetic ketoacidosis incidence among children with new-onset type 1 diabetes in Poland and its association with COVID-19 outbreak—two-year cross-sectional national observation by PolPeDiab Study Group. Pediatr Diabetes. 2022;23(7):944-955. doi:10.1111/pedi.13379 35700323 PMC9350002

[51] Raicevic M, Samardzic M, Soldatovic I, Curovic Popovic N, Vukovic R. Trends in nationwide incidence of pediatric type 1 diabetes in Montenegro during the last 30 years. Front Endocrinol (Lausanne). 2022;13:991533. doi:10.3389/fendo.2022.991533 36147568 PMC9485557

[52] Vlad A, Serban V, Timar R, . Increased incidence of type 1 diabetes during the COVID-19 pandemic in Romanian children. Medicina (Kaunas). 2021;57(9):973. doi:10.3390/medicina57090973 34577896 PMC8470921

[53] Vorgučin I, Savin M, Stanković Đ, . Incidence of type 1 diabetes mellitus and characteristics of diabetic ketoacidosis in children and adolescents during the first two years of the COVID-19 pandemic in Vojvodina. Medicina (Kaunas). 2022;58(8):1013. doi:10.3390/medicina58081013 36013479 PMC9415410

[54] Wolf RM, Noor N, Izquierdo R, . Increase in newly diagnosed type 1 diabetes in youth during the COVID-19 pandemic in the United States: a multi-center analysis. Pediatr Diabetes. 2022;23(4):433-438. doi:10.1111/pedi.13328 35218124 PMC9115477

[55] Australian Institute of Health and Welfare. Incidence of insulin-treated diabetes in Australia. February 8, 2022. Accessed December 21, 2022. https://www.aihw.gov.au/reports/diabetes/incidence-of-insulin-treated-diabetes/contents/incidence-of-insulin-treated-diabetes-in-australia

[56] Caetano FB, Lanca A, Rodrigues C, . Impact of COVID-19 in new-onset type 1 diabetes mellitus in a large Portuguese pediatric diabetes center. *Revista Portuguesa De Endocrinologia Diabetes e Metabolismo*. 2022;17(3-4):97-101.

[57] Ansar A, Livett T, Beaton W, Carrel AL, Bekx MT. Sharp rise in new-onset pediatric diabetes during the COVID-19 pandemic. WMJ. 2022;121(3):177-180. 36301642

[58] Gesuita R, Rabbone I, Marconi V, . Trends and cyclic variation in the incidence of childhood type 1 diabetes in two Italian regions over 33 years and during the COVID-19 pandemic. Diabetes Obes Metab. 2023;25(6):1698-1703. doi:10.1111/dom.15024 36810862

[59] Giorda CB, Gnavi R, Tartaglino B, . Increased incidence of type 1 diabetes in 2 years of COVID-19 pandemic. Acta Diabetol. 2023;60(4):587-589. doi:10.1007/s00592-022-01986-w 36527501 PMC9759038

[60] Matsuda F, Itonaga T, Maeda M, Ihara K. Long-term trends of pediatric type 1 diabetes incidence in Japan before and after the COVID-19 pandemic. Sci Rep. 2023;13(1):5803. doi:10.1038/s41598-023-33037-x 37037893 PMC10085994

[61] Messaaoui A, Hajselova L, Tenoutasse S. New-onset type 1 diabetes in children and adolescents before and during COVID-19 pandemic in Belgium. Pediatr Diabetes. 2022;23:54.

[62] Sasidharan Pillai S, Has P, Quintos JB, . Incidence, severity, and presentation of type 2 diabetes in youth during the first and second year of the COVID-19 pandemic. Diabetes Care. 2023;46(5):953-958. doi:10.2337/dc22-1702 36637859

[63] van den Boom L, Kostev K, Kuss O, Rathmann W, Rosenbauer J. Type 1 diabetes incidence in children and adolescents during the COVID-19 pandemic in Germany. Diabetes Res Clin Pract. 2022;193:110146. doi:10.1016/j.diabres.2022.110146 36347421 PMC9637016

[64] Knip M, Parviainen A, Turtinen M, ; Finnish Pediatric Diabetes Register. SARS-CoV-2 and type 1 diabetes in children in Finland: an observational study. Lancet Diabetes Endocrinol. 2023;11(4):251-260. doi:10.1016/S2213-8587(23)00041-4 36958868

[65] Caetano FB, Lanca A, Rodrigues C, et al. Risk factors for diabetic ketoacidosis through eight years of new-onset type 1 diabetes mellitus in a large Portuguese pediatric diabetes center: a shift towards younger age. Pediatr Diabetes. 2021;22(suppl 30):86-87.

[66] Rücker G, Schwarzer G, Carpenter JR, Schumacher M. Undue reliance on I(2) in assessing heterogeneity may mislead. BMC Med Res Methodol. 2008;8:79. doi:10.1186/1471-2288-8-79 19036172 PMC2648991

[67] Qeadan F, Tingey B, Egbert J, . The associations between COVID-19 diagnosis, type 1 diabetes, and the risk of diabetic ketoacidosis: a nationwide cohort from the US using the Cerner Real-World Data. PLoS One. 2022;17(4):e0266809. doi:10.1371/journal.pone.0266809 35439266 PMC9017888

[68] Kamrath C, Rosenbauer J, Tittel SR, . Frequency of autoantibody-negative type 1 diabetes in children, adolescents, and young adults during the first wave of the COVID-19 pandemic in Germany. Diabetes Care. 2021;44(7):1540-1546. doi:10.2337/dc20-2791 33990377

[69] Kuitunen I, Artama M, Mäkelä L, Backman K, Heiskanen-Kosma T, Renko M. Effect of social distancing due to the COVID-19 pandemic on the incidence of viral respiratory tract infections in children in Finland during early 2020. Pediatr Infect Dis J. 2020;39(12):e423-e427. doi:10.1097/INF.0000000000002845 32773660

[70] Nygren M, Carstensen J, Koch F, Ludvigsson J, Frostell A. Experience of a serious life event increases the risk for childhood type 1 diabetes: the ABIS population-based prospective cohort study. Diabetologia. 2015;58(6):1188-1197. doi:10.1007/s00125-015-3555-2 25870022

[71] Abela AG, Fava S. Why is the incidence of type 1 diabetes increasing? Curr Diabetes Rev. 2021;17(8):e030521193110. doi:10.2174/1573399817666210503133747 33949935

[72] Rewers M, Ludvigsson J. Environmental risk factors for type 1 diabetes. Lancet. 2016;387(10035):2340-2348. doi:10.1016/S0140-6736(16)30507-4 27302273 PMC5571740

[73] Mathieu E, Rodés-Guirao L, Appel C, . Coronavirus pandemic (COVID-19): our world in data. Accessed December 21, 2022. https://ourworldindata.org/coronavirus

[74] Duca LM, Reboussin BA, Pihoker C, . Diabetic ketoacidosis at diagnosis of type 1 diabetes and glycemic control over time: the SEARCH for Diabetes in Youth study. Pediatr Diabetes. 2019;20(2):172-179. doi:10.1111/pedi.12809 30556249 PMC6361710

[75] Patterson CC, Dahlquist G, Harjutsalo V, . Early mortality in EURODIAB population-based cohorts of type 1 diabetes diagnosed in childhood since 1989. Diabetologia. 2007;50(12):2439-2442. doi:10.1007/s00125-007-0824-8 17901942

[76] Birkebaek NH, Kamrath C, Grimsmann JM, . Impact of the COVID-19 pandemic on long-term trends in the prevalence of diabetic ketoacidosis at diagnosis of paediatric type 1 diabetes: an international multicentre study based on data from 13 national diabetes registries. Lancet Diabetes Endocrinol. 2022;10(11):786-794. doi:10.1016/S2213-8587(22)00246-7 36202118 PMC9597608

[77] Kamrath C, Rosenbauer J, Eckert AJ, . Incidence of COVID-19 and risk of diabetic ketoacidosis in new-onset type 1 diabetes. Pediatrics. 2021;148(3):e2021050856. doi:10.1542/peds.2021-050856 34011636

[78] Macy ML, Smith TL, Cartland J, Golbeck E, Davis MM. Parent-reported hesitancy to seek emergency care for children at the crest of the first wave of COVID-19 in Chicago. Acad Emerg Med. 2021;28(3):355-358. doi:10.1111/acem.14214 33475211 PMC8013472

[79] Kyeremateng R, Oguda L, Asemota O; International Society for Social Pediatrics and Child Health (ISSOP) COVID-19 Working Group. COVID-19 pandemic: health inequities in children and youth. Arch Dis Child. 2022;107(3):297-299. doi:10.1136/archdischild-2020-320170 33574027

[80] Dragano N, Dortmann O, Timm J, . Association of household deprivation, comorbidities, and COVID-19 hospitalization in children in Germany, January 2020 to July 2021. JAMA Netw Open. 2022;5(10):e2234319. doi:10.1001/jamanetworkopen.2022.34319 36190730 PMC9530965

[81] Saatci D, Ranger TA, Garriga C, . Association between race and COVID-19 outcomes among 2.6 million children in England. JAMA Pediatr. 2021;175(9):928-938. doi:10.1001/jamapediatrics.2021.1685 34152371 PMC8218232

[82] Koripalli M, Giruparajah M, Laur C, Shulman R. Selecting an intervention to prevent ketoacidosis at diabetes diagnosis in children using a behavior change framework. Pediatr Diabetes. 2022;23(3):406-410. doi:10.1111/pedi.13314 35001490

