# Supplementary material for: Incidence of Diabetes in Children and Adolescents During the COVID-19 Pandemic: A Systematic Review and Meta-Analysis
Source: JAMA Netw Open. 2023 Jun 30;6(6):e2321281. doi: 10.1001/jamanetworkopen.2023.21281 (PMC10314307; doi:10.1001/jamanetworkopen.2023.21281)
Supplement: Supplement 1. — eTable 1. Subject Database and Gray Literature Search Strategies eTable 2. Risk-of-Bias Evaluation Criteria Domains eTable 3. Risk-of-Bias Assessments for Included Studies, Using the ROBINS-E Tool eFigure. Rate Ratios Reported in the Meta-analysis by Rahmati et al, by Length of the Pandemic Observation Period [file jamanetwopen-e2321281-s001.pdf]

Supplementary Online Content

D'Souza D, Empringham J, Pechlivanoglou P, Uleryk EM, Cohen E, Shulman R. Incidence of diabetes in children and adolescents during the COVID-19 pandemic: a systematic review and meta-analysis. *JAMA Netw Open*. 2023;6(6):e2321281. doi:10.1001/jamanetworkopen.2023.21281

eTable 1. Subject Database and Gray Literature Search Strategies

eTable 2. Risk-of-Bias Evaluation Criteria Domains

eTable 3. Risk-of-Bias Assessments for Included Studies, Using the ROBINS-E Tool

eFigure. Rate Ratios Reported in the Meta-analysis by Rahmati et al, by Length of the Pandemic Observation Period

This supplementary material has been provided by the authors to give readers additional information about their work.

© 2023 D'Souza D et al. *JAMA Network Open*.

eTable 1. Subject database and Grey Literature search strategies

Listed below are the individual subject database strategies search from 2020 to March 28, 2023. The grey Literature sources are listed by Jurisdiction and contain some notes on retrieval.

MEDLINE:

Ovid MEDLINE: Epub Ahead of Print, In-Process & Other Non-Indexed Citations, Ovid MEDLINE® Daily and Ovid MEDLINE® <1946-Present>

#	Searches	Results	Comment
1	exp Coronavirus/	164029	
2	exp Coronavirus Infections/	227264	
3	(coronavirus* or corona virus* or OC43 or NL63 or 229E or HKU1 or HCoV* or ncov* or covid* or sars-cov* or sarscov* or Sars-coronavirus* or Severe Acute Respiratory Syndrome Coronavirus*).mp.	365087	
4	COVID-19.rx,px,ox. or severe acute respiratory syndrome coronavirus 2.os.	1822	
5	COVID-19/	215801	
6	"COVID-19 Vaccines"/	19033	
7	covid-19 testing/ or covid-19 serological testing/ or covid-19 nucleic acid testing/	10758	
8	((pneumonia or covid* or coronavirus* or corona virus* or ncov* or 2019-ncov or sars*).mp. or exp pneumonia/) and Wuhan.mp.	7929	
9	(2019-ncov or ncov19 or ncov-19 or 2019-novel CoV or sars-cov2 or sars-cov-2 or sarscov2 or sarscov-2 or Sars-coronavirus2 or Sars-coronavirus-2 or SARS-like coronavirus* or coronavirus-19 or covid19 or covid-19 or covid 2019 or ((novel or new or nouveau) adj2 (CoV or nCoV or covid or coronavirus* or corona virus or Pandemi*2)) or ((covid or covid19 or covid-19) and pandemic*2) or (coronavirus* and pneumonia)).mp.	346313	
10	COVID-19.rx,px,ox. or severe acute respiratory syndrome coronavirus 2.os.	1822	
11	or/1-10	370706	Covid search terms
12	11 and 20191201:20301231.(dt).	351112	date specific Covid 19 results
13	diabetes mellitus, type 1/	85218	
14	((brittle or autoimmune or "juvenile-onset" or (juvenile adj2 onset) or (insulin adj2 dependent) or "insulin-dependent" or (ketosis adj2	120184	

© 2023 D’Souza D et al. *JAMA Network Open*.

	prone) or "ketosis-prone" or (sudden adj2 onset) or "sudden-onset" or "type 1" or "type i" or iddm) adj3 diabet*).mp.		
15	Diabetic Ketoacidosis/ or ketoacidosis.mp.	12414	
16	or/13-15	127973	Type 1 Diabetes terms
17	12 and 16	1123	Base clinical set - Type 1 diabetes and Covid
18	Diabetes Mellitus, Type 2/	167870	
19	(diabet* or niddm).mp.	828242	
20	(HbA1c or A1c).mp.	60204	
21	(glucose adj2 (monitor* or level* or intolerance* or test or tests or tested or testing)).mp.	133597	
22	(metabolic adj2 (control* or monitor*)).mp.	17135	
23	hypoglycemic agents/ or acetohexamide/ or biguanides/ or biphasic insulins/ or buformin/ or butoxamine/ or carbutamide/ or chlorpropamide/ or exenatide/ or gliclazide/ or glipizide/ or glyburide/ or insulin/ or insulin aspart/ or insulin detemir/ or insulin glargine/ or insulin lispro/ or insulin, isophane/ or insulin, lente/ or insulin, long-acting/ or insulin, regular, pork/ or insulin, short-acting/ or insulin, ultralente/ or isophane insulin, human/ or linagliptin/ or liraglutide/ or metformin/ or nateglinide/ or phenformin/ or pioglitazone/ or rosiglitazone/ or sitagliptin phosphate/ or tolazamide/ or tolbutamide/ or topiramate/ or troglitazone/ or vildagliptin/ or glycoside hydrolase inhibitors/ or acarbose/ or amylin receptor agonists/ or islet amyloid polypeptide/ or dipeptidyl-peptidase iv inhibitors/ or sodium-glucose transporter 2 inhibitors/ or canagliflozin/	282409	
24	(hypoglycemic or acetohexamide or biguanides or buformin or butoxamine or carbutamide or chlorpropamide or exenatide or gliclazide or glipizide or glyburide or insulin or insulins or linagliptin or liraglutide or metformin or nateglinide or phenformin or pioglitazone or rosiglitazone or "sitagliptin phosphate" or tolazamide or tolbutamide or topiramate or troglitazone or vildagliptin or "glycoside hydrolase inhibitor*" or acarbos or "amylin receptor agonist*" or "islet amyloid polypeptide*" or "dipeptidyl-peptidase iv inhibitor*" or "sodium-glucose transporter 2 inhibitor*" or canagliflozin).mp.	547179	
25	or/18-24	1163830	Type 2 diabetes terms
26	11 and 25	11565	Base set 2 - Type 2 Diabetes and Covid

© 2023 D'Souza D et al. *JAMA Network Open*.

27	limit 26 to ("all child (0 to 18 years)" or "young adult (19 to 24 years)")	1209	Age group limit
28	(infan* or newborn* or new-born* or perinat* or neonat* or baby* or babies or toddler* or minor* or boy or boys or boyfriend or boyhood or girl* or kid or kids or child* or schoolchild* or adolescen* or juvenil* or youth* or teen* or under*age* or pubescen* or pediatric* or paediatric* or peadiatric* or prematur* or preterm* or school*).mp.	5427928	Paediatric textword terms
29	27 or (26 and 28)	1877	Type 2 Diabetes paediatric results
30	17 or 29	2517	Combined results
31	limit 30 to english language	2468	Language limit
32	morbidity/ or incidence/ or prevalence/	642360	Incidence terms
33	("new-onset" or (new adj2 onset*) or onset or incident* or incidence* or (delayed adj2 diagnos*) or presentation* or (new adj2 (case* or present* or diagnos*)) or prevalence or prevalent or occurrence* or epidemiolog* or variance* or morbidit*).ti,ab,kf.	4046511	Incidence textword terms
34	(sn or ep).fs.	2722501	floating subheadings statistic-numbers or epidemiology
35	or/32-34	5667222	All incidence terms
36	31 and 35	1736	Incidence specific subset of Final Results

EMBASE

Embase <1980 to 2023 Week 12>

#	Searches	Results	Comment
1	exp coronavirinae/	116146	
2	coronavirus infection/ or severe acute respiratory syndrome/	22321	
3	(coronavirus* or corona virus* or OC43 or NL63 or 229E or HKU1 or HCoV* or ncov* or covid* or sars-cov* or sarscov* or Sars-coronavirus* or Severe Acute Respiratory Syndrome Coronavirus*).mp.	463958	
4	coronavirus disease 2019/ or asymptomatic coronavirus disease 2019/ or exp covid-19 skin manifestation/ or covid-19-associated acute hemorrhagic necrotizing encephalopathy/ or covid-19-associated coagulopathy/ or covid-19-associated	337562	

© 2023 D’Souza D et al. *JAMA Network Open*.

	meningoencephalitis/ or covid-19-associated nephropathy/ or long covid/ or pediatric multisystem inflammatory syndrome/		
5	sars-cov-2 vaccine/ or anti-sars-cov-2 agent/ or abdavomeran/ or "ad26.cov2.s vaccine"/ or ag0302-covid19 vaccine/ or bbibp-corv vaccine/ or bnt 162 vaccine/ or comirnaty/ or convidicea/ or coronavac/ or covaxin/ or elasomeran/ or epivaccorona/ or ganulameran/ or nvx-cov2373 vaccine/ or pidacmeran/ or pittsburgh coronavirus vaccine/ or reluscovtogene ralaplasmid/ or "sputnik light (vaccine)"/ or sputnik v vaccine/ or vaxzevria/ or wibp-corv vaccine/ or zifivax/ or zorecimeran/	31280	
6	covid-19 testing/ or covid-19 nucleic acid testing/ or covid-19 serological testing/	9152	
7	((pneumonia or covid* or coronavirus* or corona virus* or ncov* or 2019-ncov or sars*).mp. or exp pneumonia/) and Wuhan.mp.	9963	
8	(2019-ncov or ncov19 or ncov-19 or 2019-novel CoV or sars-cov2 or sars-cov-2 or sarscov2 or sarscov-2 or Sars-coronavirus2 or Sars-coronavirus-2 or SARS-like coronavirus* or coronavirus-19 or covid19 or covid-19 or covid 2019 or ((novel or new or nouveau) adj2 (CoV or nCoV or covid or coronavirus* or corona virus or Pandemi*2)) or ((covid or covid19 or covid-19) and pandemic*2) or (coronavirus* and pneumonia)).mp.	405967	
9	or/1-8	473040	Covid search terms
10	insulin dependent diabetes mellitus/ or latent autoimmune diabetes in adults/	138766	
11	((brittle or autoimmune or "juvenile-onset" or (juvenile adj2 onset) or (insulin adj2 dependent) or "insulin-dependent" or (ketosis adj2 prone) or "ketosis-prone" or (sudden adj2 onset) or "sudden-onset" or "type 1" or "type i" or iddm) adj3 diabet*).mp.	461766	
12	Diabetic Ketoacidosis/ or ketoacidosis.mp.	25587	
13	or/10-12	474907	Type 1 Diabetes terms
14	9 and 13	6763	Base clinical set - Type 1 diabetes and Covid
15	diabetes mellitus/ or impaired glucose tolerance/ or non insulin dependent diabetes mellitus/	949257	
16	(diabet* or niddm).mp.	1396398	
17	(HbA1c or A1c).mp.	168374	

© 2023 D’Souza D et al. *JAMA Network Open*.

18	(glucose adj2 (monitor* or level* or intolerance* or test or tests or tested or testing)).mp.	416868	
19	(metabolic adj2 (control* or monitor*)).mp.	24455	
20	antidiabetic agent/ or acetohexamide/ or biguanide derivative/ or biguanide/ or buformin/ or butoxamine/ or carbutamide/ or chlorpropamide/ or exp exendin 4/ or gliclazide/ or glipizide/ or glibenclamide/ or biphasic insulin/ or insulin/ or insulin aspart/ or insulin aspart plus insulin degludec/ or insulin defalan/ or insulin degludec/ or insulin degludec plus liraglutide/ or insulin detemir/ or insulin efsitora alfa/ or insulin glargine/ or insulin glargine plus lixisenatide/ or insulin glulisine/ or insulin icodec/ or insulin lispro/ or insulin peglispro/ or insulin sensitizing agent/ or insulin tregopil/ or insulin zinc suspension/ or liraglutide/ or pig insulin/ or metformin/ or metformin 4 chlorophenoxyacetate/ or metformin embonate/ or metformin glycinate/ or metformin plus pioglitazone/ or metformin plus repaglinide/ or metformin plus rosiglitazone/ or metformin plus saxagliptin/ or metformin plus sitagliptin/ or metformin plus vildagliptin/ or phenformin/ or oral antidiabetic agent/ or linagliptin/ or nateglinide/ or phenformin/ or pioglitazone/ or rosiglitazonesitagliptin/ or topiramate/ or troglitazone/ or vildagliptin/ or glycosidase inhibitor/ or acarbose/ or amylin/ or amylin receptor agonist/	542256	
21	exp dipeptidyl peptidase IV inhibitor/	26683	
22	sodium glucose cotransporter 2 inhibitor/ or canagliflozin/ or canagliflozin plus metformin/	14822	
23	(hypoglycemic or acetohexamide or biguanides or buformin or butoxamine or carbutamide or chlorpropamide or exenatide or gliclazide or glipizide or glyburide or insulin or insulins or linagliptin or liraglutide or metformin or nateglinide or phenformin or pioglitazone or rosiglitazone or "sitagliptin phosphate" or tolazamide or tolbutamide or topiramate or troglitazone or vildagliptin or "glycoside hydrolase inhibitor*" or acarbos or "amylin receptor agonist*" or "islet amyloid polypeptide*" or "dipeptidyl-peptidase iv inhibitor*" or "sodium-glucose transporter 2 inhibitor*" or canagliflozin).mp.	1013835	
24	or/15-23	1919201	Type 2 diabetes terms
25	9 and 24	30596	Base set 2 - Type 2 Diabetes and Covid
26	limit 25 to (infant <to one year> or child <unspecified age> or preschool child <1 to 6 years> or school child <7 to 12 years> or adolescent <13 to 17 years>)	2408	Age group limit
27	(infan* or newborn* or new-born* or perinat* or neonat* or baby* or babies or toddler* or minor* or boy or boys or boyfriend or boyhood or girl* or kid or kids or child* or schoolchild* or adolescen* or juvenil* or	5582727	

© 2023 D’Souza D et al. *JAMA Network Open*.

	youth* or teen* or under*age* or pubescen* or pediatric* or paediatric* or peadiatric* or prematur* or preterm* or school*).mp.		
28	26 or (25 and 27)	4443	Type2 diabetes and children
29	14 or 28	9854	Combined Type 1 and paediatric Type 2 results
30	morbidity/ or incidence/ or prevalence/	1767886	
31	("new-onset" or (new adj2 onset*) or onset or incident* or incidence* or (delayed adj2 diagnos*) or presentation* or (new adj2 (case* or present* or diagnos*)) or prevalence or prevalent or occurrence* or epidemiolog* or variance* or morbidit*).ti,ab,kf. [***textword suggestions****]	5575115	
32	(statistic* or epidemiolog*).ti,ab,kf.	2559718	floating subheading substitutes in EMBASE
33	or/30-32	7303180	incidence terms
34	29 and 33	4757	Incidence specific subset of Final Results
35	limit 34 to (english language and yr="2020 -Current")	4635	FINAL results

Cochrane

March 28, 2023

ID	Search	Hits	
#1	MeSH descriptor: [Coronavirus] explode all trees	2204	
#2	MeSH descriptor: [Coronavirus Infections] explode all trees	4555	
#3	coronavirus* or corona virus* or OC43 or NL63 or 229E or HKU1 or HCoV* or ncov* or covid* or sars-cov* or sarscov* or "Sars-coronavirus*" or (Severe NEAR/2 Acute NEAR/2 Respiratory NEAR/2 Syndrome NEAR/2 Coronavirus*)	16963	
#4	"COVID-19"	14731	
#5	MeSH descriptor: [COVID-19 Vaccines] explode all trees	378	
#6	MeSH descriptor: [COVID-19 Testing] explode all trees	97	
#7	pneumonia or covid* or coronavirus* or corona virus* or ncov* or "2019-ncov" or sars*	35574	

© 2023 D'Souza D et al. *JAMA Network Open*.

#8	MeSH descriptor: [Pneumonia] explode all trees	8466	
#9	(#7 or #8) and wuhan	353	
#10	"2019-ncov" or ncov19 or "ncov-19" or "2019-novel CoV" or "sars-cov2" or "sars-cov-2" or sarscov2 or "sarscov-2" or "Sars-coronavirus2" or "Sars-coronavirus-2" or "SARS-like coronavirus*" or "coronavirus-19" or covid19 or "covid-19" or "covid 2019"	15155	
#11	((novel or new or nouveau) NEAR/2 (CoV or nCoV or covid or coronavirus* or corona virus or Pandemi*2)) or ((covid or covid19 or "covid-19") and pandemic*2) or (coronavirus* and pneumonia)	2393	
#12	{or #1-#6, #9-#11}	17073	Covid search terms
#13	MeSH descriptor: [Diabetes Mellitus, Type 1] explode all trees	6707	
#14	((brittle or autoimmune or "juvenile-onset" or (juvenile NEAR/2 onset) or (insulin NEAR/2 dependent) or "insulin-dependent" or (ketosis NEAR/2 prone) or "ketosis-prone" or (sudden NEAR/2 onset) or "sudden-onset" or "type 1" or "type i" or iddm) NEAR/3 diabet*)	32232	
#15	MeSH descriptor: [Diabetic Ketoacidosis] this term only	176	
#16	ketoacidosis	1209	
#17	{or #13-#16}	32741	Type 1 Diabetes terms
#18	#12 and #17	180	Base clinical set - Type 1 diabetes and Covid
#19	MeSH descriptor: [Diabetes Mellitus, Type 2] explode all trees	22821	
#20	diabet* or niddm	117352	
#21	HbA1c or A1c	27714	
#22	(glucose NEAR/2 (monitor* or level* or intolerance* or test or tests or tested or testing))	34813	
#23	(metabolic adj2 (control* or monitor*))	386	
#24	MeSH descriptor: [Hypoglycemic Agents] explode all trees	10406	
#25	hypoglycemic or acetohexamide or biguanides or buformin or butoxamine or carbutamide or chlorpropamide or exenatide or gliclazide or glipizide or glyburide or insulin or insulins or	82641	

© 2023 D’Souza D et al. *JAMA Network Open*.

	linagliptin or liraglutide or metformin or nateglinide or phenformin or pioglitazone or rosiglitazone or "sitagliptin phosphate" or tolazamide or tolbutamide or topiramate or troglitazone or vildagliptin or "glycoside hydrolase inhibitor*" or acarbos or "amylin receptor agonist*" or "islet amyloid polypeptide*" or "dipeptidyl-peptidase iv inhibitor*" or "sodium-glucose transporter 2 inhibitor*" or canagliflozin		
#26	{or #19-#25}	152655	Type 2 diabetes terms
#27	#12 and #26	1284	Base set 2 - Type 2 Diabetes and Covid
#28	(infan* or newborn* or new-born* or perinat* or neonat* or baby* or babies or toddler* or minor* or boy or boys or boyfriend or boyhood or girl* or kid or kids or child* or schoolchild* or adolescen* or juvenil* or youth* or teen* or under*age* or pubescen* or pediatric* or paediatric* or peadiatric* or prematur* or preterm* or school*)	481465	Age group limit
#29	#27 and #28	576	
#30	#18 or #29	679	Type2 diabetes and children
#31	MeSH descriptor: [Morbidity] this term only	999	Combined Type 1 and paediatric Type 2 results
#32	MeSH descriptor: [Incidence] this term only	12583	
#33	MeSH descriptor: [Prevalence] this term only	6477	
#34	"new-onset" or (new NEAR/2 onset*) or onset or incident* or incidence* or (delayed NEAR/2 diagnos*) or presentation* or (new NEAR/2 (case* or present* or diagnos*)) or prevalence or prevalent or occurrence* or epidemiolog* or variance* or morbidit*	413819	
#35	{or #31-#34}	413819	incidence terms
#36	#30 and #35	495	Incidence specific subset of Final Results

© 2023 D’Souza D et al. *JAMA Network Open*.

Scopus

(TITLE-ABS-KEY (covid) AND TITLE-ABS-KEY (diabete* OR a1c OR hba1c OR hypoglycemic OR acetohexamide OR biguanides OR buformin OR butoxamine OR carbutamide OR chlorpropamide OR exenatide OR gliclazide OR glipizide OR glyburide OR insulin OR insulins OR linagliptin OR liraglutide OR metformin OR nateglinide OR phenformin OR pioglitazone OR rosiglitazone OR "sitagliptin phosphate" OR tolazamide OR tolbutamide OR topiramate OR troglitazone OR vildagliptin OR "glycoside hydrolase inhibitor*" OR acarbos OR "amylin receptor agonist*" OR "islet amyloid polypeptide*" OR "dipeptidyl-peptidase iv inhibitor*" OR "sodium-glucose transporter 2 inhibitor*" OR canagliflozin OR ketoacidos*) AND TITLE-ABS-KEY (infan* OR newborn* OR new-born* OR perinat* OR neonat* OR baby* OR babies OR toddler* OR minor* OR boy OR boys OR boyfriend OR boyhood OR girl* OR kid OR kids OR child* OR schoolchild* OR adolescen* OR juvenil* OR youth* OR teen* OR under*age* OR pubescen* OR pediatric* OR paediatric* OR peadiatric* OR prematur* OR preterm* OR school*) AND TITLE-ABS-KEY (((new W/2 onset*) OR onset OR incident* OR incidence* OR (delayed W/2 diagnos*) OR presentation* OR (new W/2 (case* OR present* OR diagnos*)) OR prevalence OR prevalent OR occurrence* OR epidemiolog* OR variance* OR morbidit*))) AND (LIMIT-TO (LANGUAGE , "English")) and pub years 2020-2023 Results: 1815 ref

Web of Science

Run on March 28, 2023

Set	Query	Results	Comment
**#1**	TS=(coronavirus* or (corona NEAR/2 virus*) or OC43 or NL63 or 229E or HKU1 or HCoV* or ncov* or covid* or sars-cov* or sarscov* or "Sars-coronavirus*" or (Severe NEAR/2 Acute NEAR/2 Respiratory NEAR/2 Syndrome NEAR/2 Coronavirus*))	446,902	
**#2**	(TS=(coronavirus* or (corona NEAR/2 virus*) or OC43 or NL63 or 229E or HKU1 or HCoV* or ncov* or covid* or sars-cov* or sarscov* or "Sars-coronavirus*" or (Severe NEAR/2 Acute NEAR/2 Respiratory NEAR/2 Syndrome NEAR/2 Coronavirus*))) AND TS=(("2019-ncov" or ncov19 or "ncov-19" or "2019-novel CoV" or "sars-cov2" or "sars-cov-2" or sarscov2 or "sarscov-2" or "Sars-coronavirus2" or "Sars-coronavirus-2" or "SARS-like coronavirus*" or "coronavirus-19" or covid19 or "covid-19" or "covid 2019" or ((novel or new or nouveau) NEAR/2 (CoV or nCoV or covid or coronavirus* or "corona virus" or Pandemi*2)) or ((covid or covid19 or "covid-19") and pandemic*2) or (coronavirus* and pneumonia)))	416,060	
**#3**	#1 or #2	446,902	Covid terms
**#4**	TS=((diabetesNEAR/2 mellitus NEAR/2 "type 1") or ((brittle or autoimmune or "juvenile-onset" or (juvenile NEAR/2 onset) or (insulin NEAR/2 dependent) or "insulin-dependent" or (ketosis NEAR/2 prone) or "ketosis-prone" or (sudden NEAR/2 onset) or "sudden-	119,567	Diabetes Type 1 terms

© 2023 D'Souza D et al. *JAMA Network Open*.

	onset" or "type 1" or "type i" or iddm) NEAR/3 diabet*) or ketoacidosis)		
**#5**	#3 and #4	1,188	Base set 1 – Covid and type 1 diabetes
**#6**	TS=((diabet* or niddm or HbA1c or A1c or (glucose NEAR/2 (monitor* or level* or intolerance* or test or tests or tested or testing)) or (metabolic adj2 (control* or monitor*))))	1,003,694	
**#7**	TS=(hypoglycemic or acetohexamide or biguanides or buformin or butoxamine or carbutamide or chlorpropamide or exenatide or gliclazide or glipizide or glyburide or insulin or insulins or linagliptin or liraglutide or metformin or nateglinide or phenformin or pioglitazone or rosiglitazone or "sitagliptin phosphate" or tolazamide or tolbutamide or topiramate or troglitazone or vildagliptin or "glycoside hydrolase inhibitor*" or acarbos or "amylin receptor agonist*" or "islet amyloid polypeptide*" or "dipeptidyl-peptidase iv inhibitor*" or "sodium-glucose transporter 2 inhibitor*" or canagliflozin)	640,517	
**#8**	#6 OR #7	1,351,034	Diabetes type 2 terms
**#9**	#3 and #8	11,711	Base set 2 – Covid and type 2 diabetes
**#10**	TS=(infan* or newborn* or new-born* or perinat* or neonat* or baby* or babies or toddler* or minor* or boy or boys or boyfriend or boyhood or girl* or kid or kids or child* or schoolchild* or adolescen* or juvenil* or youth* or teen* or under*age* or pubescen* or pediatric* or paediatric* or peadiatric* or prematur* or preterm* or school*)	5,142,810	Paediatric age group terms
**#11**	#9 and #10	1,315	Base set 2 – limit to paediatrics
**#12**	#5 OR #11	2,061	Combined Base set 1 or 2 results
**#13**	Refined by publication years 2020, 2021, 2022, 2023 English language	2,061	Final results – publication year and language limits

Grey Literature sources:

Search terms (Covid and Diabetes and (statistics or incidence).

© 2023 D'Souza D et al. *JAMA Network Open*.

Diabetes specific organizations/associations

Retrieved N=11

Statistical information N=1

Disease Associations	Data-year reported	URLs
CAPACIty registry (Canada)	No stats	https://bcchr.ca/news/first-canadian-national-pediatric-diabetes-registry-equity-diversity-inclusion
CoviDiab Project (King's College , London and Monash University)	No stats	https://covidiab.e-dendrite.com/
Commonwealth Fund (New York)	No stats	https://www.commonwealthfund.org/international-health-policy-center/system-stats
European Association for the Study of Diabetes	No stats	https://www.easd.org/statements.html
Diabetes Canada	No Stats	https://www.diabetes.ca/managing-my-diabetes/tools---resources/registry
Diabetes Canada – Nova Scotia	No stats	https://www.nshealth.ca/diabetes
Diabetes Canada – Prince Edward Island	No stats	https://www.princeedwardisland.ca/en/topic/diabetes
Diabetes Canada - Newfoundland and Labrador	2019	https://www.diabetes.ca/media/DiabetesCharter
Diabetes Canada – New Brunswick	2020	https://diabetes.ca/DiabetesCanadaWebsite/media/Advocacy-and-Policy/Backgrounder/2020_Backgrounder_New-Brunswick_English_FINAL.pdf
Oslo Diabetes Research Centre (Norway)	No stats	https://www.oslodiabetes.no/projects
Sweet Project	In progress	https://www.sweet-project.org/sweet-database.php

Government Webpages

© 2023 D'Souza D et al. *JAMA Network Open*.

Hand screened for statistics on incidence of Type 1 diabetes for years 2020+

Retrieved N=32

Statistical information N=12

Government	Data-year reported	URLs
World health Organization (WHO)	2021	https://www.euro.who.int/__data/assets/pdf_file/0003/505371/registries-information-systems-diabetes-consultation-eng.pdf
EuroHealthNet	No stats	https://eurohealthnet.eu/publication/health-highlights/
European Union/European Commission	2020	https://ec.europa.eu/health/state-health-eu/country-health-profiles_en#country-health-profiles-2021
Danish Health Data - The National Diabetes Register	No stats	https://www.danishhealthdata.com/find-health-data/Det-Nationale-Diabetesregister
Denmark National Institute of Public Health	No stats	https://www.sst.dk/en/English/publications?searchWord=diabetes
Denmark (Public Health Agency)	No Stats	https://www.sst.dk/en/English/publications?searchWord=diabetes
Finnish Institute for health and welfare	No Stats	https://thl.fi/en/web/thlfi-en/research-and-development/research-and-projects/diabetes-in-finland-findm-
Netherlands – Diabetes Mellitus	2021	https://www.vzinfo.nl/diabetes-mellitus/leeftijd-en-geslacht
Netherlands. National Institute for Public Health and the Environment Ministry of Health, Welfare and Sport	No Stars	https://www.rivm.nl/en/search?search=diabetes&f%5B0%5D=sdv_published%3A2020-03&f%5B1%5D=sdv_published%3A2020-05&f%5B2%5D=sdv_published%3A2020-10&f%5B3%5D=sdv_published%3A2021-02&f%5B4%5D=sdv_published%3A2021-04&f%5B5%5D=sdv_published%3A2021-06&f%5B6%5D=sdv_published%3A2021-08&f%5B7%5D=sdv_published%3A2022-01&f%5B8%5D=sdv_published%3A2022-02&f%5B9%5D=sdv_published%3A2022-03
The Norwegian Childhood Diabetes Registry	No stats	https://www.oslodiabetes.no/childhood
Norwegian Institute of Public Health	2017	https://www.fhi.no/en/mp/chronic-diseases/diabetes/
Norwegian Public Health Association	No Stats	https://eupha.org/norwegian-public-health-association

© 2023 D'Souza D et al. *JAMA Network Open*.

Oslo Diabetes Research Centre (Norway)	No stats	https://www.oslodiabetes.no/projects
Swedish National Diabetes Register (NDR)	No Stats	https://www.ndr.nu/#/Research
Sweden - Annual Diabetes report 2020 + (Swediabkids)	2020	https://www.ndr.nu/pdfs/Annual_Report_Swediabkids_2020.pdf
Public Health Agency of Sweden	2019	https://www.folkhalsomyndigheten.se/folkhalsorapportering-statistik/statistik-a-o/ovrig-statistik-a-o/diabetes/?t=county<br><br>http://fohm-app.folkhalsomyndigheten.se/Folkhalsodata/pxweb/sv/A_Folkhalsodata/A_Folkhalsodata__Z_ovrigdata__HLVkn__Hals/diabet.px/table/tableViewLayout1/
Australian Institute of Health and Welfare	2020<br><br>2020	https://www.aihw.gov.au/reports/diabetes/incidence-of-insulin-treated-diabetes/data<br><br>https://www.aihw.gov.au/reports/diabetes/incidence-of-insulin-treated-diabetes/contents/methods-and-classifications
Australian Institute of Health and Welfare. The National (insulin-treated) Diabetes Register 2021; Quality Statement<br><br>NDSS diabetes data statistics	2021<br><br>2022	https://meteor.aihw.gov.au/content/index.phtml/itemId/753745<br><br>https://www.ndss.com.au/about-the-ndss/diabetes-facts-and-figures/diabetes-data-snapshots/
National Association of Diabetes Centres (Australia)	2020	https://nadc.net.au/past-anda/
New Zealand Virtual Diabetes Register	2021	https://www.health.govt.nz/our-work/diseases-and-conditions/diabetes/about-diabetes/virtual-diabetes-register-vdr
New Zealand. Ministry of Health (Web Tool interactive)	2021	https://minhealthnz.shinyapps.io/virtual-diabetes-register-web-tool/
Canada Data Blog	2016	https://health-infobase.canada.ca/datalab/diabetes-blog.html
Canada New Brunswick Department of Health	2011	https://www2.gnb.ca/content/gnb/en/departments/health/patientinformation/PrimaryHealthCare/A-Comprehensive-Diabetes-Strategy-for-New-Brunswickers.html

© 2023 D'Souza D et al. *JAMA Network Open*.

Canada New Brunswick Department of Public Health,	No Stats	https://www2.gnb.ca/content/dam/gnb/Departments/h-s/pdf/en/Publications/Profiles/ProfilesHealthDiabetes.pdf
Canada Public Health Ontario	2020	https://www.publichealthontario.ca/en/Data-and-Analysis/Chronic-Disease/Chronic-Disease-Hospitalization
Canada Manitoba Health	2017/2018	https://www.gov.mb.ca/health/quickstats/disease.html
Canada Saskatchewan Department of Health	No Stats	https://www.saskatchewan.ca/residents/health/diseases-and-conditions/diabetes
Canada Alberta Department of Health	No Stats	https://myhealth.alberta.ca/Health/Pages/conditions.aspx?hwid=center1010
Canada British Columbia Department of Health	No Stats	https://www2.gov.bc.ca/gov/content/health/practitioner-professional-resources/bc-guidelines/diabetes
United Stated. Centers for Disease Control and Prevention Diabetes Statistical Reports (Incidence of Newly Diagnosed Diabetes)	2015	https://www.cdc.gov/diabetes/data/statistics-report/index.html
CDC. Diabetes Report Card. Diabetes in Youth	2019	https://www.cdc.gov/diabetes/library/reports/reportcard/diabetes-in-youth.html
United States. Centers for Disease Control and Prevention. Diabetes Statistics	2020	https://www.niddk.nih.gov/health-information/health-statistics#diabetes

© 2023 D’Souza D et al. *JAMA Network Open*.

eTable 2. Risk of bias evaluation criteria domains[1]

Domain	Evaluating Questions
Domain 1: Risk of bias due to confounding	1. Did the authors control for all the important confounding factors for which this was necessary?<br>2. Were confounding factors that were controlled for (and for which control was necessary) measured validly and reliably by the variables available in this study?<br>3. Did the authors control for any variables after the start of the exposure period being studied that could have been affected by the exposure?<br>4. Did the use of negative controls, or other considerations, suggest serious uncontrolled confounding?
Domain 2: Risk of bias arising from measurement of the exposure	1. Does the measured exposure well-characterize the exposure metric specified to be of interest in this study?<br>2. Was the exposure likely to be measured with error, or misclassified?<br>3. Could mismeasurement or misclassification of exposure have been differential (i.e. related to the outcome or risk of the outcome)?<br>4. Is non-differential measurement error likely to bias the estimated effect of exposure on outcome?
Domain 3: Risk of bias in selection of participants into the study (or into the analysis)	1. Did follow-up begin at (or close to) the start of the exposure window for most participants?<br>2. Is the effect of exposure likely to be constant over the period of follow up analysed?<br>3. Was selection of participants into the study (or into the analysis) based on participant characteristics observed after the start of the exposure window being studied?<br>4. Were these characteristics likely to be influenced by exposure or a cause of exposure?<br>5. Were these characteristics likely to be influenced by the outcome or a cause of the outcome?<br>6. Is it likely that the analysis corrected for all of the potential selection biases identified in 3.1 and 3.2 above?<br>7. Did sensitivity analyses demonstrate that the likely impact of the potential selection biases identified in 3.1 or 3.2 above was minimal?
Domain 4: Risk of bias due to post-exposure interventions	1. Were there post-exposure interventions that were influenced by prior exposure during the follow-up period?<br>2. Is it likely that the analysis corrected for the effect of post-exposure interventions that were influenced by prior exposure?
Domain 5: Risk of bias due to missing data	1. Were complete data on exposure status available for all, or nearly all, participants?<br>2. Were complete data on the outcome available for all, or nearly all, participants?<br>3. Were complete data on confounding variables available for all, or nearly all, participants?<br>4. Is the result based on a complete case analysis?<br>5. Was exclusion from the analysis because of missing data (in exposure, confounders or the outcome) likely to be related to the true value of the outcome?

© 2023 D'Souza D et al. *JAMA Network Open*.

	6. Were all or most predictors of missingness (in exposure, confounders or the outcome) included in the analysis model?<br>7. Was the analysis based on inputting missing values?<br>8. Was imputation performed appropriately?<br>9. Was an appropriate alternative method used to correct for bias due to missing data?<br>10. Is there evidence that the result was not biased by missing data?
Domain 6: Risk of bias arising from measurement of the outcome	1. Could measurement or ascertainment of the outcome have differed between exposure groups or levels of exposure?<br>2. Were outcome assessors aware of study participants' exposure history?<br>3. Could assessment of the outcome have been influenced by knowledge of participants' exposure history?
Domain 7: Risk of bias in selection of the reported result	1. Was the result reported in accordance with an available, pre-determined analysis plan?<br>2. Is the reported effect estimate likely to be selected, based on desirability of the magnitude (or statistical significance) of the estimated effect of exposure on outcome, from multiple exposure measurements within the exposure domain?<br>3. Is the reported effect estimate likely to be selected, based on desirability of the magnitude (or statistical significance) of the estimated effect of exposure on outcome, from multiple outcome measurements within the outcome domain?<br>4. Is the reported effect estimate likely to be selected, based on desirability of the magnitude (or statistical significance) of the estimated effect of exposure on outcome, from multiple analyses of the exposure-outcome relationship?<br>5. Is the reported effect estimate likely to be selected, based on the basis of desirability of the results (e.g. statistical significance), from different subgroups?
Overall risk of bias	A. Low risk of bias except for concerns about uncontrolled confounding<br>B. Some concerns<br>C. High risk of bias<br>D. Very high risk of bias

1. Adapted from ROBINS-E Development Group (Higgins J, Morgan R, Rooney A, Taylor K, Thayer K, Silva R, Lemeris C, Akl A, Arroyave W, Bateson T, Berkman N, Demers P, Forastiere F, Glenn B, Hróbjartsson A, Kirrane E, LaKind J, Luben T, Lunn R, McAleenan A, McGuinness L, Meerpohl J, Mehta S, Nachman R, Obbagy J, O'Connor A, Radke E, Savović J, Schubauer-Berigan M, Schwingl P, Schunemann H, Shea B, Steenland K, Stewart T, Straif K, Tilling K, Verbeek V, Vermeulen R, Viswanathan M, Zahm S, Sterne J). Risk Of Bias In Non-randomized Studies - of Exposure (ROBINS-E). Launch version, 1 June 2022. Available from: https://www.riskofbias.info/welcome/robins-e-tool

© 2023 D'Souza D et al. *JAMA Network Open*.

eTable 3. Risk of bias assessments for included studies, using the ROBINS-E tool.

Source	Domain 1	Domain 2	Domain 3	Domain 4	Domain 5	Domain 6	Domain 7	Overall
Alexandre, 2021	some	some	low	low	low	low	low	some
Boboc, 2021	some	some	low	low	low	low	low	some
Dilek, 2021	some	some	low	low	low	low	low	some
Kostopoulou, 2021	some	some	some	low	low	low	low	some
Mameli, 2021	some	some	low	low	low	low	low	some
Marks, 2021	some	some	low	low	low	low	low	some
Mohamed Haniffa, 2021	some	some	low	low	low	low	low	some
Moon, 2021	some	some	low	low	low	low	low	some
Vlad, 2021	some	some	low	low	low	low	low	some
Al-Abdulrazzaq, 2022	some	some	low	low	low	low	low	some
Al-Qahtani, 2022	some	some	low	low	low	low	low	some
Alassaf, 2022	some	some	low	low	low	low	low	some
Ansar, 2022	some	some	low	low	low	low	low	some
Australian Institute of Health and Welfare, 2022*	N/A	N/A	N/A	N/A	N/A	N/A	N/A	N/A
Branco Caetano, 2022	some	some	low	low	low	low	low	some
Cinek, 2022	some	some	low	low	low	low	low	some
Citron, 2022	some	some	low	low	low	low	low	some
DeLacey, 2022	some	some	low	low	low	low	low	some
Donbaloglu, 2022	some	some	low	low	low	low	low	some
Kamrath, 2022	some	some	low	low	low	low	low	some
Gottesman, 2022	some	some	low	low	low	low	low	some

© 2023 D’Souza D et al. *JAMA Network Open*.

Source	Domain 1	Domain 2	Domain 3	Domain 4	Domain 5	Domain 6	Domain 7	Overall
Guo, 2022	some	some	low	low	low	low	low	some
Kaya, 2022	some	some	low	low	low	low	low	some
Leiva-Gea, 2022	some	some	low	low	low	low	low	some
Magge, 2022	some	some	low	low	low	low	low	some
Messaaoui, 2022	some	some	low	low	low	low	low	some
Modarelli, 2022	some	some	low	low	low	low	low	some
Passanisi, 2022	some	some	low	low	low	low	low	some
Pietrzak, 2022	some	some	low	low	low	low	low	some
Raicevic, 2022	some	some	low	low	low	low	low	some
Reschke, 2022	some	some	low	low	low	low	low	some
Schiaffini, 2022	some	some	low	low	low	low	low	some
Schmitt, 2022	some	some	low	low	low	low	low	some
Shulman, 2022	some	some	low	low	low	low	low	some
van den Boom, 2022	some	some	low	low	low	low	low	some
Vorgucin, 2022	some	some	low	low	low	low	low	some
Wolf, 2022	some	some	low	low	low	low	low	some
Baechle, 2023	some	some	low	low	low	low	low	some
Gesuita, 2023	some	some	low	low	low	low	low	some
Giorda, 2023	some	some	low	low	low	low	low	some
Matsuda, 2023	some	some	low	low	low	low	low	some
Sasidharan Pillai, 2023	some	some	low	low	low	low	low	some

*This record could not be assessed as this was a grey literature record in the form of a national registry.

© 2023 D'Souza D et al. *JAMA Network Open*.

eFigure 1. Rate ratios reported in Rahmati et al. 2022 meta-analysis by the length of their pandemic observation period

Source	RR (95% CI)
**Length = <3 months**	
Atlas, 2021	0.65 [0.47; 0.90]
Dzygalo, 2020	0.65 [0.43; 1.00]
Jacob, 2021	1.07 [0.80; 1.44]
Kamrath, 2020	1.06 [0.97; 1.15]
Lawrence, 2021	1.22 [0.51; 2.95]
Rabbon, 2020	0.77 [0.63; 0.95]
Total	0.94 [0.87; 1.01]
Heterogeneity: $\chi^2_5 = 18.85$ ($P = .002$), $I^2 = 73\%$	
**Length = 3–6 months**	
Alaqeel, 2021	0.72 [0.48; 1.07]
Goldman, 2022	1.29 [1.01; 1.65]
McGlacken−Byrne, 2021	0.57 [0.31; 1.03]
Unsworth, 2020	2.00 [1.08; 3.72]
Hawkes, 2022	0.79 [0.58; 1.08]
Sellers, 2021	1.10 [0.92; 1.31]
Ho, 2021	0.94 [0.72; 1.22]
Vlad, 2021	1.17 [0.53; 2.57]
Salmi, 2022	1.47 [1.05; 2.06]
Total	1.06 [0.96; 1.18]
Heterogeneity: $\chi^2_8 = 22.54$ ($P = .004$), $I^2 = 65\%$	
**Length = 12 months**	
Mameli, 2021	1.11 [0.93; 1.32]
Boboc, 2021	1.30 [1.02; 1.66]
Kostopoulou, 2021	1.24 [0.65; 2.34]
Modarelli, 2022	1.48 [0.94; 2.34]
Al−Abdulrazzaq, 2021	1.07 [0.91; 1.25]
Dilel, 2021	1.61 [1.11; 2.32]
Gottesman, 2022	1.57 [1.25; 1.98]
Herrero, 2022	1.61 [0.96; 2.71]
Lee, 2021	1.00 [0.42; 2.40]
Marks, 2021	1.15 [0.93; 1.43]
Total	1.22 [1.13; 1.33]
Heterogeneity: $\chi^2_9 = 13.23$ ($P = .15$), $I^2 = 32\%$	

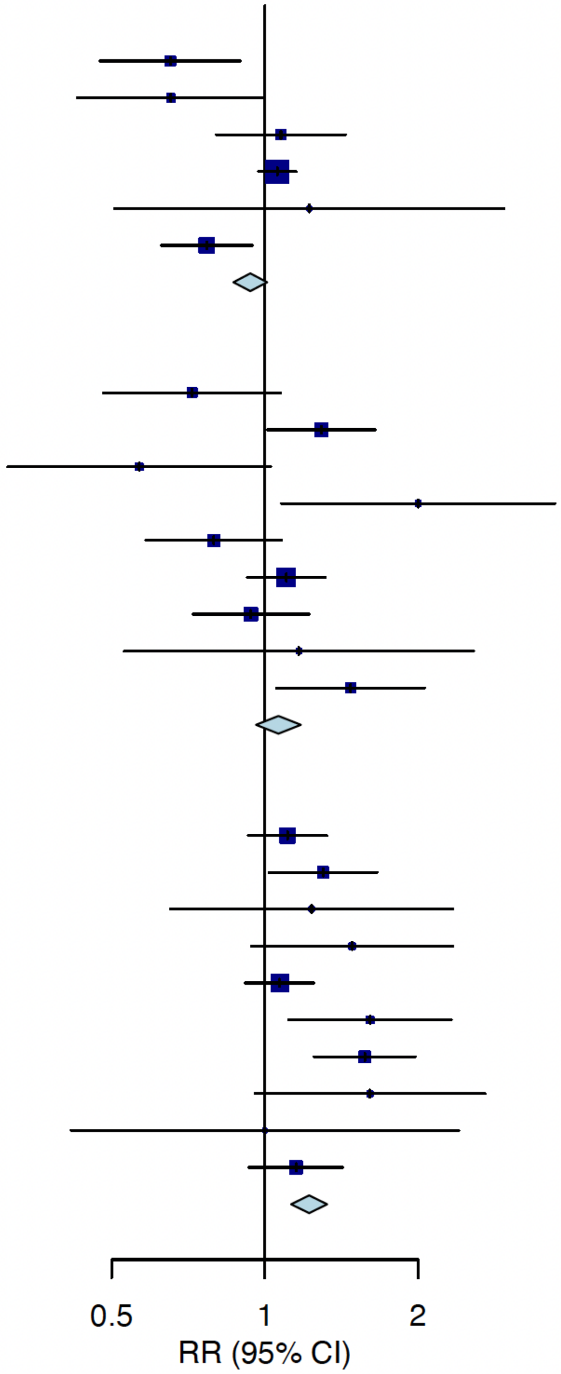

Heterogeneity: $\chi^2_{24} = 70.11$ ($P < .001$), $I^2 = 66\%$
Test for subgroup differences: $\chi^2_2 = 21.83$ ($P < .001$)

© 2023 D'Souza D et al. *JAMA Network Open*.
