# Supplementary material for: Incidence of Diabetes in Children and Adolescents During the COVID-19 Pandemic: A Systematic Review and Meta-Analysis
Source: JAMA Netw Open. 2023 Jun 30;6(6):e2321281. doi: 10.1001/jamanetworkopen.2023.21281 (PMC10314307; doi:10.1001/jamanetworkopen.2023.21281)
Supplement: Supplement 2. — Data Sharing Statement [file jamanetwopen-e2321281-s002.pdf]

Data Sharing Statement

D'Souza. Incidence of Diabetes in Children and Adolescents During the COVID-19 Pandemic. *JAMA Netw Open*. Published June 30, 2023. doi:10.1001/jamanetworkopen.2023.21281

Data
Data available: Yes
Data types: Other (please specify)
Additional Information: data will be made available to researchers whose proposed use of the data has been approved
How to access data: rayzel.shulman@sickkids.ca
When available: With publication

Supporting Documents
Document types: None

Additional Information
Who can access the data: researchers whose proposed use of the data has been approved
Types of analyses: for research purposes
Mechanisms of data availability: with investigator support and after approval of a proposal
